# Piceid Octanoate Protects Retinal Cells against Oxidative Damage by Regulating the Sirtuin 1/Poly-ADP-Ribose Polymerase 1 Axis In Vitro and in rd10 Mice

**DOI:** 10.3390/antiox13020201

**Published:** 2024-02-04

**Authors:** Seyed Mohamadmehdi Moshtaghion, Estefanía Caballano-Infantes, Álvaro Plaza Reyes, Lourdes Valdés-Sánchez, Patricia Gallego Fernández, Berta de la Cerda, Maurizio S. Riga, Manuel Álvarez-Dolado, Pablo Peñalver, Juan C. Morales, Francisco J. Díaz-Corrales

**Affiliations:** 1 Department of Integrative Pathophysiology and Therapies, Andalusian Molecular Biology and Regenerative Medicine Centre (CABIMER), Junta de Andalucía, CSIC, Universidad de Sevilla, Universidad Pablo de Olavide, Avda. Américo Vespucio 24, 41092 Seville, Spain; mehdi.moshtaghion@cabimer.es (S.M.M.); alvaro.plaza@cabimer.es (Á.P.R.); lourdes.valdes@cabimer.es (L.V.-S.); patriciagallegofernandez22@gmail.com (P.G.F.); berta.delacerda@cabimer.es (B.d.l.C.); maurizio.riga@cabimer.es (M.S.R.); manuel.alvarez@cabimer.es (M.Á.-D.); 2 Department of Biochemistry and Molecular Pharmacology, Institute of Parasitology and Biomedicine López-Neyra (IPBLN), PTS-Granada, Avda. del Conocimiento, 17, 18016 Granada, Spain; pablo@ipb.csic.es (P.P.); jcmorales@ipb.csic.es (J.C.M.)

**Keywords:** retinal degeneration, retinitis pigmentosa, oxidative stress, mitochondrial dysfunction, reactive oxygen species (ROS), parthanatos, polyphenols, SIRT1, PARP1, (NAD^+^/NADH ratio), rd10 mice

## Abstract

Retinitis pigmentosa is a common cause of inherited blindness in adults, which in many cases is associated with an increase in the formation of reactive oxygen species (ROS) that induces DNA damage, triggering Poly-ADP-Ribose Polymerase 1 (PARP1) activation and leading to parthanatos-mediated cell death. Previous studies have shown that resveratrol (RSV) is a promising molecule that can mitigate PARP1 overactivity, but its low bioavailability is a limitation for medical use. This study examined the impact of a synthesized new acylated RSV prodrug, piceid octanoate (PIC-OCT), in the 661W cell line against H_2_O_2_ oxidative stress and in rd10 mice. PIC-OCT possesses a better ADME profile than RSV. In response to H_2_O_2_, 661W cells pretreated with PIC-OCT preserved cell viability in more than 38% of cells by significantly promoting SIRT1 nuclear translocation, preserving NAD^+^/NADH ratio, and suppressing intracellular ROS formation. These effects result from expressing antioxidant genes, maintaining mitochondrial function, reducing PARP1 nuclear expression, and preventing AIF nuclear translocation. In rd10 mice, PIC-OCT inhibited PAR-polymer formation, increased SIRT1 expression, significantly reduced TUNEL-positive cells in the retinal outer nuclear layer, preserved ERGs, and enhanced light chamber activity (all *p* values < 0.05). Our findings corroborate that PIC-OCT protects photoreceptors by modulating the SIRT1/PARP1 axis in models of retinal degeneration.

## 1. Introduction

Inherited retinal diseases (IRDs) are a clinically and genetically heterogeneous group of eye pathologies mainly characterized by progressive degeneration or dysfunction of retinal cells, causing vision loss and ultimately leading to blindness [1]. Retinitis pigmentosa (RP) is one of the most common IRDs, affecting approximately 1 in 4000 people worldwide. Most RP patients present initially progressive degeneration of rod photoreceptors, followed by the loss of cones and retinal pigment epithelium (RPE) cells. Night blindness is the earliest symptom, usually starting in adolescence, followed by a narrowing of the visual field and reduced amplitude of electroretinogram (ERG) waves. Mutations in more than 80 different genes cause non-syndromic RP, and most of them encode retinal-specific proteins, but others are ubiquitously expressed [2,3,4]. Given the high genetic heterogeneity of RP, it is crucial to understand the common cell death mechanisms to develop novel therapies that can effectively treat RP independently of the disease-causing mutant genes [5].

Apoptosis has been commonly associated with photoreceptor cell death in IRDs; however, recent studies suggest that parthanatos, a programmed cell death mechanism independent of caspases, is also involved in several retinal degenerative diseases that produce blindness, including RP [6,7,8,9]. Parthanatos is a process initiated by recruiting poly-ADP-ribose polymerase 1 (PARP1) at the nuclear level, triggered by DNA damage. PARP1 is a DNA damage sensor that uses oxidized nicotinamide adenine dinucleotide (NAD^+^) as a substrate for its ADP-ribosylation activity [10]. Excessive activation of this DNA repair mechanism increases the synthesis of poly (ADP-ribose) (PAR) polymers, which promote the mitochondrial release and nuclear translocation of the apoptosis-inducing factor (AIF), leading to chromatin condensation, large-scale DNA fragmentation, and ultimately cell death [8,11]. In addition, PARP1 overactivation may affect cellular energy production due to the large consumption of NAD^+^ [12,13,14]. It has been reported that PARP1 is overactivated in different RP mouse models, suggesting that parthanatos may contribute to photoreceptor cell death in RP independently of the disease-causing mutant genes [5,11]. In addition, cell death significantly decreased in PARP1 knockout mouse retina explants treated with a selective PDE6 (photoreceptor phosphodiesterase) inhibitor to induce retinal degeneration. These results support the hypothesis that excessive activation of PARP1 plays an essential role in retinal degeneration and opens the door to the search for new drugs that could protect photoreceptors through the inhibition of parthanatos [8].

Since parthanatos is characterized by the overactivation of PARP1, specific inhibitors of this DNA repair protein have been tested to avoid photoreceptor degeneration [15,16,17]. PARP1 inhibitors have been primarily used for cancer therapy because they can prevent the activation of DNA repair mechanisms and produce the death of neoplastic cells [18]. Olaparib was the first PARP1 inhibitor approved by the Food and Drug Administration (FDA) to treat metastatic breast cancer [18]. This drug has also shown a neuroprotective effect delaying the progressive loss of photoreceptors in the *Pde6b* mutant rd1 mice [15]. Other PARP1 inhibitors, such as BMN-673, 3-aminobenzamide, and PJ34, have also shown neuroprotective effects in retinal cells [16,17]. However, the chronic and systemic use of PARP1 inhibitors has risky adverse effects that could interfere with the repurposing of these types of drugs to treat RP [19]. In this context, developing new PARP1 inhibitors may be an excellent strategy to treat or prevent photoreceptor degeneration and other neurodegenerative diseases [20].

**Figure 1:**
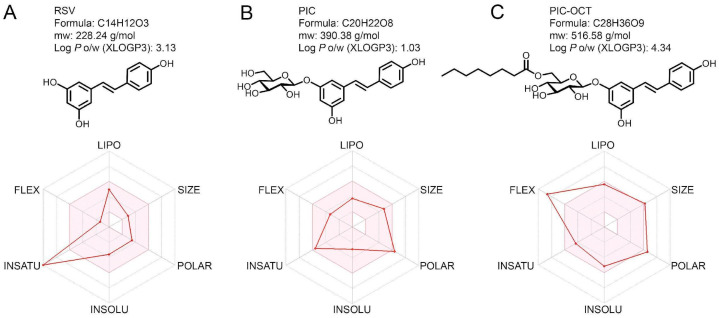
PIC-OCT drug-likeness evaluation. Resveratrol (RSV) (**A**), piceid (PIC) (**B**), and piceid octanoate (PIC-OCT) (**C**) bioavailability radars are shown. The formulas and molecular weight (mw) values of RSV, PIC, and PIC-OCT are also displayed. The bioavailability radars, indicated by the pink zones, evaluate the drug-likeness of each molecule. The optimum range for each property is shown: lipophilicity (LIPO): XLOGP3 between −0.7 and +5.0; size (SIZE): mw between 150 and 500 g/mol; polarity (POLAR): TPSA among 20 and 130Å2; solubility (INSOLU): log S not higher than 6; saturation (INSATU): fraction of carbons in the sp3 hybridization not less than 0.25; and flexibility (FLEX): no more than 9 rotatable bonds. PIC-OCT shows an acceptable drug-likeness except for flexibility and polarity. The Lipophilicity XLOGP3 value was higher in PIC-OCT than in RSV and PIC.

One molecule that could act as a PARP1 modulator is the natural polyphenol 3,5,4 -trans-trihydroxystilbene (resveratrol, RSV) (Figure 1A). It has been reported that RSV treatment decreased PARP1 levels in an in vitro model of photoreceptor injury [21]. Moreover, RSV has also shown a therapeutic effect in neurodegenerative disease models [21,22,23,24,25,26]. In this context, it has been observed that RSV induces specific alterations in the microglia transcriptome, suppressing inflammatory pathways and safeguarding against photoreceptor apoptosis mediated by microglia [27]. Therefore, the therapeutic effect of RSV might not just be restricted to neuronal cells. The exact mechanism by which RSV could modulate PARP1 expression in photoreceptors remains to be understood. RSV, like other natural polyphenols, can reduce cellular oxidative stress [28], therefore reducing oxidative DNA damage. On the other hand, RSV is a sirtuin 1 (SIRT1) activator, a class III NAD^+^-dependent histone deacetylase [29,30,31]. Hence, SIRT1 and PARP1 establish functional connectivity through their utilization of a common cofactor, NAD^+^, and common substrates that can interact antagonistically to modulate gene transcription and post-translational modification of several proteins involved in DNA repair, metabolism, and inflammation [12,13,14,32,33,34]. Recent research indicates that these proteins actively engage in shared pathways, delicately offering cells an environment to balance survival and apoptosis [35].

RSV is known to be one of the natural polyphenols with the highest activity as a SIRT1-activating compound (SATC) [30,36,37,38]. The mechanism by which RSV activates SIRT1 seems to be related to allosteric modifications of SIRT1, increasing its enzymatic activity. However, RSV could also act indirectly on some kinases, such as AMPK, increasing the synthesis of NAD^+^. SIRT1 modulation is a therapeutic target in neurodegeneration, and hence, SATCs have the potential to be innovative treatments for retinal degenerative diseases [32,39]. Given their current exploration as therapeutic targets across diverse conditions, including cancer, metabolic disorders, and neurodegenerative models, an in-depth investigation of the mutual pathways they navigate holds significant promise for enhanced understanding of the SIRT1/PARP1 axis [35].

Regarding the potential benefits of SIRT1 activation, its participation in the modulation of the downstream pathways of the antioxidant defense system has been described, such as catalase activity, regulation of metabolic balance, clearance of unwanted unfolded proteins, modulation of neuroinflammation, and promotion of axonal growth and dendritic branching [40,41,42]. Despite the neuroprotective potential of RSV as a SIRT1 activator and PARP1 inhibitor, the low oral bioavailability of this polyphenol has limited its use as an effective drug to treat neurodegenerative diseases [43]. Furthermore, because RSV shows very poor solubility in water and is rapidly metabolized and eliminated from the organism, high doses must be administered, potentially resulting in toxic side effects [44,45]. For these reasons, new molecules that can improve the pharmacokinetic properties of RSV are needed.

To this end, Peñalver et al. designed and synthesized a library of acylated RSV derivatives by incorporating structural modifications that improved drug bioavailability and antioxidant and anti-inflammatory properties [24,46]. Piceid (PIC), or Polydatin, is a natural stilbenoid polyphenol, a glycoside derivative of RSV (Figure 1B) [24]. The neuroprotective effect of these RSV and PIC derivatives was evaluated in a zebrafish model of neuronal damage [24]. The new PIC derivative 3-O-(6′-O-octanoyl)-β-D-glucopyranoside resveratrol (PIC-OCT, Figure 1C) notably improved the acetylcholinesterase activity in zebrafish treated with pentylenetetrazole and showed higher neuroprotective effect than RSV. In a mouse model of Huntington’s disease (HD), PIC-OCT delayed the onset and reduced the severity of HD-like symptoms by enhancing locomotor activity and protecting against weight loss [24]. Furthermore, among other PIC and RSV derivatives, PIC-OCT demonstrated efficacy in delaying photoreceptor degeneration in rd10 mice [47]. However, the exact mechanism by which PIC-OCT protects the retina in this RP model remains unknown.

Regarding the current therapeutic strategies to treat RP, gene therapy is showing promising results because of its efficacy and safety [48]. Only one gene therapy approach has been approved to treat a group of IRD patients with mutations in the RPE65 gene. Just 2% of RP cases are estimated to be caused by RPE65 mutations [49], and the remaining mutations associated with RP have no treatments. This RPE65 gene augmentation therapeutic strategy (voretigene neparvovec) uses adeno-associated viral (AAV) vectors to deliver the transgene in the retina [50,51]. For AAV administration, most clinical trials of gene therapy to treat RP use subretinal injections for vector delivery; however, this route of administration can cause several side effects, such as retinal detachment, vitreous hemorrhage, and inflammation, among others. In addition, several studies have reported an antibody response and ocular inflammation stemming from immune cell infiltration after retinal gene therapy using AAV vectors [52]. Therefore, new pharmacological therapies are currently needed to mitigate the pro-inflammatory environment typically induced after subretinal administration of gene therapy, contributing to the prolonged survival of photoreceptors. Among the various pharmacotherapies available, some studies have emphasized the protective effects of natural polyphenols in slowing down the degeneration of cones in individuals with RP [53,54,55].

In this work, we study the protective activity and the mechanism of action of a new synthesized polyphenolic molecule, PIC-OCT, in an in vitro model of retinal cell degeneration and in rd10 mice. Moreover, we conduct studies to elucidate the complex mechanisms that control the interaction between the nucleus and mitochondria in an oxidative injury model. Our study demonstrates the potential of this PIC derivative as a treatment for IRDs, such as RP, due to its antioxidant properties and activity as a SIRT1/PARP1 axis modulator. PIC-OCT might prove beneficial either as a standalone therapy for RP or a complementary treatment alongside gene therapy approaches.

## 2. Materials and Methods

### 2.1. Synthesis of 3-O-(6′-O-octanoyl)-β-D-glucopyranoside Resveratrol (Piceid Octanoate, PIC-OCT)

Synthesis of piceid octanoate was previously reported by Larrosa et al. [46]. Briefly, PIC-OCT was prepared via enzymatic acylation of piceid using Novozym 435, lipase immobilized on macroporous acrylic resin. The reaction was carried out in tert-butyl alcohol (10 mL for 1 g of piceid) by adding vinyl octanoate (15 mL, 3 eq). The mixture was stirred in an orbital shaker at 60 °C for 16 h. The enzyme was decanted and separated. The solvent was evaporated and the product was purified via flash column chromatography (CH2Cl2/MeOH from 95:5 to 85:15) to obtain the corresponding PIC-OCT as a white solid (yield 83%, purity 98.5%).

### 2.2. Pharmacokinetic Study In Silico

The pharmacokinetics, drug-likeness, and bioavailability radars of RSV, PIC, and PIC-OCT were evaluated in silico using the SwissADME web tool [56]. This web tool gives free access to robust predictive models for physicochemical properties, pharmacokinetics, and drug-likeness of small molecules. The optimum range for lipophilicity (LIPO): XLOGP3 between −0.7 and +5.0; size (SIZE): molecular weight (mw) between 150 and 500 g/mol; polarity (POLAR): TPSA among 20 and 130 Å2; solubility (INSOLU): log S not higher than 6; saturation (INSATU): fraction of carbons in the sp3 hybridization not less than 0.25; and flexibility (FLEX): no more than 9 rotatable bonds were evaluated and displayed in the pink areas of the bioavailability radars shown in Figure 1.

### 2.3. Cell Culture

The mouse retinal-derived cell line (661W) was grown in culture at 37 °C in a humid chamber with 5% CO_2_ in Dulbecco’s modified Eagle’s medium F12 (DMEM/F12; Sigma-Aldrich, St. Louis, MO, USA) supplemented with 1% penicillin/streptomycin (Sigma-Aldrich), 1% glutamine (Sigma-Aldrich), and 10% fetal bovine serum (Sigma-Aldrich). The culture medium was changed every 2 or 3 days. It is important to consider that 661W is an immortalized cone photoreceptor cell line that expresses markers of photoreceptors and also markers of retinal ganglion cells, suggesting that 661W is a retinal ganglion precursor-like cell line, which shows properties of both retinal ganglion and photoreceptor cells [57]. Oxidative stress was induced by the addition of hydrogen peroxide (H_2_O_2_). The 661W cells were treated with different concentrations of H_2_O_2_ (Sigma), ranging from 150 to 500 μM. We conducted two distinct protocols. The first protocol involved chronic exposure to a low dose of H_2_O_2_ (150 µM) over an extended period (24 h) to simulate the prolonged oxidative damage inherent in the retinal degeneration process. Additionally, we investigated the impact of our novel drug, PIC-OCT, in an acute model of oxidative damage using a high dose of H_2_O_2_ (300–500 µM). To assess the progressive effects of H_2_O_2_ exposure and elucidate the molecular mechanisms influenced by PIC-OCT, 661W cells were treated with the highest H_2_O_2_ dose and evaluated at 0, 2, 4, 6, or 8 h. The cells treated with 0.02% DMSO served as the control group.

### 2.4. Cell Viability

Cell vitality was tested using the CellTiter-Blue^®^ Cell Viability Assay (Promega Corporation, Madison, WI, USA). The cells were seeded in a 96-well plate at 1 × 10^4^ cells/well density and incubated overnight at 37 °C in 5% CO_2_. To dissolve lipophilic PIC-OCT in aqueous solutions, a primary stock of PIC-OCT was diluted in DMSO, and a second dilution was carried out in DMEM/F12 to obtain the desired concentration (10, 20, and 40 μM). The cells were pretreated with PIC-OCT or PIC (10, 20, and 40 μM) for 24 h. The next day, H_2_O_2_ was added at different concentrations of 150–500 μM, and after 6 h or 24 h, the CellTiter-Blue reagent was added to the cells and further incubated for 2 h at 37 °C, 5% CO_2_. The absorbance was measured at 490 nm with a 96-well plate reader. The quantity of the formazan product, measured using 490 nm absorbance, was directly proportional to the number of living cells in the culture. In addition, the cells were seeded in 6-well plates at 3 × 10^5^ cells/well density for cell viability and flow cytometry evaluation. The cells were pretreated with PIC-OCT and exposed or not to H_2_O_2_ as described above and were then stained using ethidium homodimer-1 and counter-stained with calcein (Live/Dead Viability/Cytotoxicity kit for mammalian cells, Thermo Fisher, Waltham, MA USA) by incubating the tissue in 0.4% ethidium homodimer, 0.125% calcein in D-PBS for 30 min (min) at 37 °C, followed by single-cell dissociation using Trypsin-EDTA 0.25% (Sigma-Aldrich) at 37 °C for 3 min and, finally, stopped with the cell culture medium containing FBS. The cell suspension was filtered through a 40 μm cell strainer and centrifuged. Cell pellets were washed twice in DPBS and, finally, the number of cells was counted using the Automated Cell Counters (CellDrop™ DeNovix Inc., Washington DC, USA) or resuspended in flow cytometry buffer (2% FBS, 1 mM EDTA in DPBS) using an LSRFortessa X-20 flow cytometer equipped with 488 nm, 561 nm, 405 nm, and 640 nm lasers (BD Biosciences, Heidelberg, Germany). Data analysis was performed using FlowJo v10.7 software (BD Biosciences).

### 2.5. Measurement SIRT1 Deacetylase Activity

SIRT1 deacetylase activity was measured using the fluorometric SIRT1 Assay Kit (CS1040; Sigma) following the manufacturer’s instructions. Briefly, the assays were performed by incubating different concentrations of PIC-OCT, including 1, 2, 4, 10, 20, and 40 µM, with SIRT1 substrate solution at 37 °C for 30 min. After the addition of the developing solution and incubation at 37 °C for 10 min, fluorescence intensity was measured at 460 nm (excitation 355 nm) using a Microplate Fluorescence Reader (Varioskan Flash, Thermo Fisher) and compared with a standard curve.

### 2.6. Measurement of NAD^+^/NADH Levels

NAD levels were measured using NAD^+^/NADH Quantification Colorimetric kits (Sigma-Aldrich, Cat# MAK037) by following the manufacturer’s instructions. Briefly, 661W cells were treated with H_2_O_2_ (500 µM, 6 h) after being pretreated with PIC-OCT (40 μM) for 24 h, following the protocol described previously. Then, the cells were collected in 400 μL of NAD^+^/NADH extraction buffer. The samples from each treatment group were split into two sets: The first was used to carry out a thermal degradation assay of NADH, followed by the enzymatic cycling assay to determine NAD^+^ content. The second set measured the total NAD (NADH plus NAD^+^) content by performing the cycling assay without thermal NADH decomposition. The resulting products were read at 450 nm, and the values were converted to absolute NAD concentrations based on the simultaneously generated NAD standard curve. The NAD^+^/NADH ratio was calculated as the percentage of (total NAD − NADH)/NADH.

### 2.7. Immunofluorescence Experiments and Mitochondrial Staining

Immunofluorescence experiments were performed in cells grown on glass coverslips in a 12-well plate at 1.5 × 10^5^ cells/well density and incubated overnight at 37 °C in 5% CO_2_. The cells were fixed in 4% PFA and then permeabilized and blocked with 2% donkey serum/PBS-T for 1 h at room temperature. Incubation was performed for 1 h at room temperature with the following primary antibodies: rabbit polyclonal anti-AIF (Invitrogen, Waltham, MA, USA, PA5-48108, 1:500), mouse anti-SIRT1 (Abcam, Cambridge, UK, ab1103041, 1:500), rabbit anti-phospho-SIRT1-47s (Cell Signaling Technology, Danvers, MA, USA, #2314, 1:500), and rabbit anti-PARP1 (Abcam, ab32138, 1:500). The cells were washed three times with PBS-T and incubated with AlexaFluor^®^ secondary antibodies (Molecular Probes, Eugene, OR, USA). Coverslips were mounted on glass slides with Vectashield mounting medium (Vector Laboratories, Mowry Ave Newark, CA, USA). The nuclei were stained with Hoechst 33342 (Thermo Fisher). Mitochondrial staining with MitoTracker Mitochondrion-selective Probes culture medium containing 100 nM of MitoTracker Red CMXRos (Molecular Probes) was added to cells grown on plates and incubated at 37 °C for 30 min. After incubation, the medium containing the MitoTracker dye was replaced with a fresh medium. Confocal images of cell coverslips were captured using a spectral confocal microscope TCS SP5 (Leica, Wetzlar, Germany) with an HCX PL APO CS 40X/1.25 OIL or Lambda blue 63 1.4 OIL objectives at 22 °C and using the software LAS AF 2.7.3. ImageJ 1.53k analysis software was used to analyze the images and measure the nuclear-integrated density. Adobe Photoshop CS5.1 software was used for the digital amplification of the images.

### 2.8. Intracellular ROS Level Measurement

Intracellular ROS production was measured using dihydroethidium (DHE) dye (Assay Kit Reactive Oxygen Species ab236206, Abcam, Cambridge, UK) in live cells, which is selective for superoxide anion and hydrogen peroxide. Briefly, cells were seeded in a 96-well microplate (1 × 10^4^ cells/well) and incubated with the DHE for 30 min at 37 °C. Cells were washed with PBS 1× and analyzed for fluorescence intensity using a Microplate Fluorescence Reader (Varioskan Flash, Thermo Fisher) at the excitation and emission wavelengths of 480–520 nm and 570 nm, respectively. Antimycin A inhibits respiration by disrupting mitochondrial electron transport at complex III [58] and was used as a positive control for ROS production in 661W. N-acetyl-l-cysteine is commonly used to inhibit ROS [59] and was included as a negative control of the kit. The level of ROS generation is represented as total DHE fluorescence intensity. Data were normalized using Hoechst 33,342 (Thermo Fisher) fluorescence intensity. 

### 2.9. Western Blot (WB)

Cells were seeded in 6-well plates at 3 × 10^5^ cells/well and cultured and treated as described above. Proteins were extracted in an ice-cold RIPA buffer containing protease and phosphatase inhibitors. Cell lysates were incubated on ice for 60 min, and the homogenates were centrifuged (19,200× *g*, 20 min at 4 °C). The supernatants were collected, the protein content was measured via Bradford protein assay (Sigma), and the samples were stored at −80 °C. Thirty micrograms of each extract were separated in a denaturing 10% SDS–PAGE gel, and the proteins were transferred to a PVDF membrane (Amersham Biosciences, Little Chalfont, UK) and blocked using Superblock Blocking buffer (Thermo Fisher Scientific, Waltham, MA USA) containing 0.1% of Tween-20 (Sigma-Aldrich) for 1 h at room temperature. The primary antibody rabbit anti-γ-Tubulin (Abcam, ab11316, 1:1000), rabbit anti-Histone H3 (Cell Signaling Technology, #4499, 1:500), mouse anti-SIRT1 (Abcam, ab1103041, 1:500), rabbit anti-phospho-SIRT1-47s (Cell Signaling Technology, #2314, 1:500) or mouse anti-PAR (Enzo Life Sciences, East Farmingdale, NY, USA, ALX-804-220-R100, 1:1000) were incubated for 1 h at room temperature. The membrane was probed with the appropriate anti-HRP-conjugated secondary antibodies for 1 h at room temperature, and the immunoreactive bands were detected in a ChemiDoc MP Imaging System and analyzed using the Image Lab Version 6.1.0 software.

### 2.10. Mitochondrial Potential Membrane Assessment

The oscillations in the mitochondrial membrane potential (ΔΨm) were evaluated using the fluorescent chemical tetraethyl benzimidazolyl-carbocyanine iodide (JC-1) with the JC-1-Mitochondrial Membrane Potential Assay kit (Abcam, Cat. No. ab113850) following the manufacturer’s protocol. The 661W cells were seeded at a density of 1 × 10^4^ cells/well and allowed to adhere overnight in a black, clear-bottom 96-well plate. Following treatment, the cells were washed once with 1× dilution buffer and then incubated with 20 µM of JC-1 dye in 1× dilution buffer for 10 min at 37 °C, protected from light. The JC-1 dye was removed, cells were washed once with 1× dilution buffer, and 100 µL of fresh 1× dilution buffer was added to each well. The red fluorescence in excitation (535 nm)/emission (590 nm) and green fluorescence excitation/emission (475 nm/530 nm) were measured using a microplate fluorescence reader (Varioskan Flash, Thermo Fisher). Background fluorescence was subtracted from the fluorescence of treated cells, and then the ratio of red (polarized) fluorescence divided by that of green (de-polarized) fluorescence was calculated as a percentage of control.

### 2.11. RNA Isolation, Reverse Transcription, PCR, and Real-Time PCR

The cells were seeded in 6-well plates at 3 × 10^5^ cells/well and cultured and treated as described above. Total RNA was extracted using the RNeasy Mini kit (Qiagen, Hilden, Germany) according to the manufacturer’s instructions. After spectrophotometric quantification of RNA using NanoDrop1000 (Thermo Fisher Scientific), reverse transcription was carried out using the cDNA QuantiTect^®^ reverse transcription kit (Qiagen, Hilden, Germany) according to the manufacturer’s instructions. cDNA amplification was performed by using 1 μg of RNA as a template. Approximately 10 ng of cDNA was used for qPCR. The qPCR was performed using TaqMan^®^ Gene Expression Real-Time qPCR assays (Life-Technologies, Carlsbad, CA, USA) according to the manufacturer’s instructions, using a Thermal Cycler C100 (Bio-Rad, Hercules, CA, USA). The average cycle threshold (CT) of fluorescence units was used to analyze the mRNA levels. mRNA levels were normalized by *Gapdh* RNA levels. Quantification was calculated as fold change in gene expression = 2(−ΔΔCT) with ΔCT = CT (specific gene) − CT(*Gapdh*), and ΔΔCT = ΔCT (specific gene in sample X) − ΔCT (specific gene in reference sample). The TaqMan gene expression assays are shown in Table 1.

### 2.12. In Vivo Experiments

B6.CXB1-*Pde6*brd10/J homozygous mice, known as retinal degeneration 10 (rd10), were used for the in vivo experiments. Rd10 mice were housed in a temperature-controlled environment (21 ± 1 °C), with a relative humidity of 55 ± 5%, a light/dark cycle 08:00–20:00, and given standard mouse chow and water ad libitum. All animal procedures complied with the Spanish and European Laboratory Animal Science Association FELASA Guide for the Care and Use of Laboratory Animals and the European Union Council Directive 2010/63/EU. Postnatal day 14 (P14) rd10 mice were anesthetized with ketamine/xylazine (80/12 mg/kg body weight) and intravitreally injected with 1 µL of 10 mM PIC-OCT diluted in 5% DMSO. Untreated rd10 mice were used as controls. Intraocular injections were performed as described previously with minor modifications [60]. To evaluate PAR-polymer expression, mice were sacrificed every other day from P14 to P26. The eyes were enucleated, and the neuroretina dissected; then, RIPA buffer was used for protein extraction and WBs were performed as described above. Full-field ERGs were recorded at P28 with a ColorDome Ganzfeld (Diagnosis LCC, Mobile, AL, USA) in dark- and light-adapted conditions using increasing flash intensities as previously described [61]. The amplitudes of a- and b-waves were measured in untreated and 10 mM PIC-OCT-treated mice. The light–dark box (LDB) test was performed essentially as previously described [62]. Briefly, the LDB consisted of two identical chambers (the light and the dark one, 240 mm × 240 mm × 240 mm each) connected with a door in the middle. The light chamber was illuminated by a lightbulb at 300 lux positioned over the center of the chamber. The mice were dark-adapted for at least 8 h and stayed in the dark chamber alone for 2 min before the test without any light stimulus. After this habituation period, the mice were allowed to explore both chambers for 5 min. Only one test per mouse was performed. After each test, the box was cleaned with ethanol 70%. The test was videotaped for offline analysis. Time spent in the light chamber (s), velocity (cm/s), and the distance moved (cm) were calculated using the EthoVision tracking system XT 15 (Noldus Technology, Wageningen, The Netherlands).

### 2.13. Histology of Mouse Retinas

Immunofluorescence was conducted on eye slides obtained from mice at various developmental stages, including P18 and P28. In summary, anesthetized animals were euthanized via cervical dislocation, and their eyes were promptly excised and fixed in ice-cold 4% paraformaldehyde (4% PFA) in phosphate-buffered saline (PBS) overnight at 4 °C. Subsequently, the fixed eyes underwent an 8 h cryoprotection at room temperature in 20% sucrose–PBS, followed by an overnight cryoprotection at 4 °C in 30% sucrose–PBS for cryotome sectioning. Serial sections, 18 mm thick, were then mounted in five parallel series and subjected to immunofluorescence, involving incubation with primary and secondary antibodies. For immunofluorescence analysis, sections were mounted with the Vectashield mounting medium containing 4’,6-diamidino-2-phenylindole (DAPI) (Vector H-1201). For histology, we used the primary antibodies anti-opsin (Abcam, Ab65695), anti-rhodopsin (Abcam, ab190307), anti-recoverin (Millipore, Burlington, MA, USA, AB5585), and anti-Brn-3a (Santa Cruz, CA, USA, SC-8429). To assess cell death, we conducted the terminal deoxynucleotidyl transferase dUTP nick and labeling (TUNEL) assay using the In Situ Cell Death Detection Kit (Roche, Basel, Switzerland) following the prescribed guidelines provided by the manufacturer. To quantify the number of nuclei in the outer nuclear layer (ONL), cryosections were subjected to staining with DAPI (Sigma-Aldrich, Madrid, Spain). A direct count of TUNEL-positive signal in the outer ONL was performed using ImageJ 1.53k analysis software. We measured the ONL area. Cell death was assessed by comparing the TUNEL-positive signal with the ONL area between the experimental groups. Seven retinal sections were analyzed per experimental group.

### 2.14. Statistical Analysis

The GraphPad Prism 9 software was used for statistical analysis. The normal distribution of the samples was evaluated using the Shapiro–Wilk test. Parametric statistics were applied to data with normal distributions: unpaired *t*-test, one-way or two-way ANOVA, followed by the appropriate post hoc test. Dunnett’s test was used to compare the effect of different doses of PIC-OCT with the control; Tukey’s test was used to compare all possible pairs of groups; and Šídák’s test was used to compare specific pairs of groups. Non-parametric statistics were applied to data with non-normal distributions: Mann–Whitney U test. A *p*-value <0.05 was considered statistically significant.

## 3. Results

### 3.1. PIC-OCT Showed High Lipophilicity in an In Silico Pharmacokinetic Profile and Drug-Likeness Evaluation

Due to PIC-OCT being an RSV acylated derivative and synthesized from PIC, we studied the drug-likeness of these molecules. Herein, we used the SwissADME web tool [56] to evaluate the drug-likeness of RSV, PIC, and PIC-OCT (Figure 1). As a result, we found that PIC-OCT shows an acceptable bioavailability profile related to properties such as lipophilicity, size, solubility, and saturation, except for flexibility and polarity (Figure 1C). High flexibility accompanied by polarity in the upper limit could hinder the oral absorption of the PIC-OCT molecule; however, PIC-OCT showed higher lipophilicity (XLOGP3: 4.34) than RSV (XLOGP3: 3.13) and PIC (XLOGP3: 1.03), which could facilitate the passage of PIC-OCT through the lipid-based cell membrane to reach the cytoplasm.

### 3.2. PIC-OCT Treatment Protects 661W Cells against Oxidative Stress Caused by H_2_O_2_ Exposure

**Figure 2:**
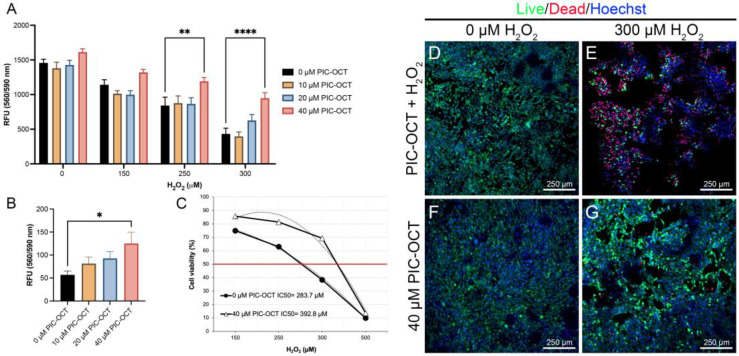
Cell viability of 661W pretreated with PIC-OCT and exposed to H_2_O_2_. The cells were grown in 96-well plates and pretreated with PIC-OCT (10–40 µM) for 24 h. Then, the 661W cells were exposed to H_2_O_2_ (150–300 µM) for 6 h with 0.02% DMSO used as a vehicle. Cell viability was measured using the CellTiter-Blue assay. Graph (**A**) shows the cell viability of the 661W cells treated with different doses of PIC-OCT and exposed to increasing doses of H_2_O_2_. The graph bars represent the mean ± SEM of relative fluorescence units (RFU) (560/590 nm) (*n* = 3). Graph (**B**) shows the cell viability of 661W cells treated with different doses of PIC-OCT and exposed to 500 µM of H_2_O_2_. PIC-OCT treatment increases the median inhibitory concentrations (IC50) of H_2_O_2_ from 283.7 µM in cells exposed to H_2_O_2_ alone to 392.8 µM in the PIC-OCT-treated group (**C**). The red line represents 50% cell viability, and the dashed lines represent the trendline (**C**). The cells were stained with calcein (**D**–**G**; green) to label live cells and counterstained with ethidium homodimer-1 (**D**–**G**; red) to label dead cells using the Live/Dead Viability/Cytotoxicity kit. Nuclei were stained with the Hoechst dye (**D**–**G**; blue). The number of live cells in the vehicle-treated (**D**; green) and PIC-OCT-treated (**F**; green) groups was similar. An amount of 300 µM of H_2_O_2_ exposure increases the number of dead cells (**E**; red), and PIC-OCT treatment protects against the toxic effect of H_2_O_2_ (**G**), evidenced by the low number of cells stained with ethidium homodimer-1 (**G**; red) and the preservation of cells labeled with calcein (**G**; green). Statistics: Two-way ANOVA followed by Dunnett’s test to compare different treated groups with the control (0 µM of PIC-OCT). * *p* < 0.05; ** *p* < 0.01; **** *p* < 0.0001. Scale bars represent 250 µm.

Given that H_2_O_2_ treatment generates reactive-induced oxidative stress affecting cell viability in other cell types, we sought to verify whether H_2_O_2_ can induce the death of photoreceptor-like cells in a dose-dependent manner. To this end, we determined the optimal concentration of H_2_O_2_ that could be used to establish an in vitro model of IRDs using the mouse retinal cone 661W cell line. To determine the protective effect of PIC-OCT, an acute H_2_O_2_ exposure protocol was tested. The 661W cells were pretreated with 10, 20, or 40 μM of PIC-OCT for 24 h and were then exposed to 150, 250, 300, or 500 μM of H_2_O_2_ for 6 h. The vehicle group was treated only with 0.02% DMSO. The cell viability was evaluated by the conversion of a redox dye in a fluorescent product as described in the materials and methods section. The group pretreated with 40 μM of PIC-OCT significantly preserved cell viability in 661W cells exposed to 250, 300, and 500 μM of H_2_O_2_ (Figure 2A,B) in a dose-dependent manner. Moreover, 661W cells were pretreated with PIC using the same concentrations tested for PIC-OCT experiments. Unlike PIC-OCT, the PIC molecule lacking octanoic acid (OCT) did not show a cytoprotective effect (Figure S1). The median inhibitory concentrations (IC50) of H_2_O_2_ in 661W cells treated with vehicle or pretreated with PIC-OCT were also estimated (Figure 2C). The IC50 of H_2_O_2_ treatment at 6 h exposure in the vehicle group was 283.7 μM. On the other hand, in the 40 μM of the PIC-OCT group, the IC50 increased to 392.8 μM, representing a protective effect on the IC50 of more than 38% in the PIC-OCT-treated group. The labeling of live and dead cells also showed an increase in dead cells in the 300 µM H_2_O_2_-exposed group (Figure 2E; red). The cytoprotective effect of 40 µM of PIC-OCT pretreatment in the H_2_O_2_-exposed group is also demonstrated by the low number of dead cells stained with ethidium homodimer-1 (Figure 2G; red) and by the preservation of the number of live cells stained with calcein (Figure 2G; green).

Cell viability was also determined using a chronic H_2_O_2_ exposure protocol. The 661W cells were seeded in 6-well culture dishes and treated with a low dose (150 µM) of H_2_O_2_ for 24 h. The cells were also pretreated with different doses of PIC-OCT ranking from 0.2 to 20 µM for 12 h (Figure S2). Then, the cells were stained with the Live/Dead Viability/Cytotoxicity kit as described in Section 2. Like in the acute H_2_O_2_ exposure protocol, PIC-OCT treatment preserved cell viability when a low dose of H_2_O_2_ was used. As expected, the number of dead cells stained with ethidium homodimer-1 increased in the group exposed to peroxide (Figure S2B′, red), while the number of live cells labeled with calcein decreased (Figure S2B′, green). The pretreatment with PIC-OCT prevented the toxic effect of H_2_O_2_, as observed in the images (Figure S2C–E′). Live and dead cells were quantified via direct cell counter equipment (Figure S2F) or cytometry (Figure S2G).

### 3.3. PIC-OCT Molecule Activates SIRT1, Preserving NAD^+^/NADH Ratio and Upregulates the Nuclear Expressions of SIRT1 and Phospho-SIRT1 (Ser47)

**Figure 3:**
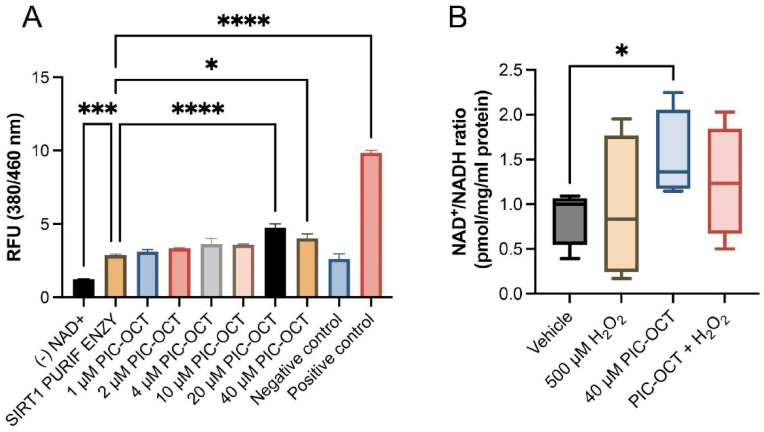
PIC-OCT activates SIRT1 and increases the reduced NAD^+^/NADH ratio. The deacetylation activity of SIRT1 was measured via the fluorescence of a specific deacetylated substrate. Graph (**A**) represents SIRT1 deacetylase activity in different concentrations of PIC-OCT including 1, 2, 4, 10, 20, and 40 µM (*n* = 3). Nicotinamide and resveratrol (RSV) were used as negative and positive controls, respectively. The group with SIRT1 purified enzyme and without PIC-OCT (SIRT1 PURIF ENZY) was used to evaluate the effect of adding different concentrations of PIC-OCT to the reaction. The group (−) NAD^+^ was used to evaluate the lack of SIRT1 activity without its coenzyme. The reduced NAD^+^/NADH ratio was measured in 661W cells treated with 500 µM of H_2_O_2_ and 40 µM of PIC-OCT as described in Section 2. The cells were collected, and the total intracellular level of NAD^+^ was measured in heated and unheated samples to estimate the NAD^+^/NADH ratio. The graph (**B**) represents the reduced NAD^+^/NADH ratio adjusted to the total amount of proteins (*n* = 4). Statistics in graph (**A**): One-way ANOVA followed by Dunnett’s test to compare different treated groups with the control (SIRT1 PURIF ENZ). * *p* < 0.05; *** *p* < 0.001; **** *p* < 0.0001. Statistics in graph (**B**): Mann–Whitney U test. * *p* < 0.05.

Once the cell viability was determined, the evaluation of the SIRT1 activity was carried out using a widely used kit that evaluates the deacetylation activity of SIRT1 using a substrate that contains an acetylated lysine side chain. The deacetylated substrate releases a highly fluorescent group, and the fluorescence is directly proportional to the deacetylation activity of the SIRT1 enzyme. Amounts of 20 and 40 µM of PIC-OCT significantly increased the activity of SIRT1, although this increase was less than that observed with the RSV positive control. Activation of SIRT1 has been described as the main mechanism of action of RSV. However, other cellular targets of RSV have been suggested, such as increased mitochondrial NAD^+^/NADH ratio due to directly stimulating NADH dehydrogenases. SIRT1 is an NAD^+^-dependent deacetylated histone, so an increase in NAD+ will increase SIRT1 activity. As NAD^+^ declines due to oxidative stress and in an age-related manner [13,63], it induces mitochondrial dysfunction and nuclear DNA damage, which may exacerbate neurodegenerative conditions. In this line, we further explore the effect of PIC-OCT on the redox balance in cells. Here, we found that PIC-OCT increased the NAD^+^/NADH ratio compared with untreated cells, indicating that PIC-OCT increased NAD^+^ levels, which might be responsible for the increased activation of SIRT1 as well (Figure 3B).

**Figure 4:**
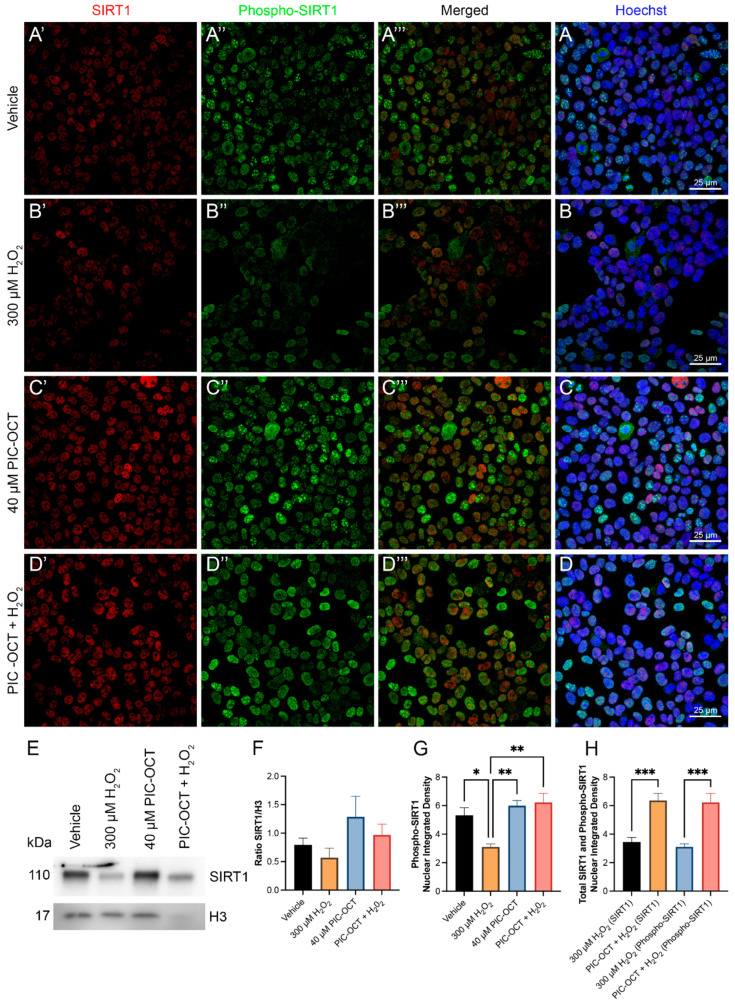
Expressions of SIRT1 and phospho-SIRT1 (Ser47) in 661W cells treated with PIC-OCT. The 661W cells were treated with 300 µM of H_2_O_2_ and 40 μM of PIC-OCT for 24 h, as described in Section 2, and then the cells were immunostained with anti-SIRT1 (**A′**–**D′**; red) and anti-phospho-SIRT1 (Ser47) antibodies (**A″**–**D″**; green). The merge of anti-SIRT1 and anti-phospho-SIRT1 (Ser47) shows the colocalization (**A‴**–**D‴**). Hoechst dye was used to visualize the cell nuclei (blue) and the merge of the 3 channels it can be observed in the panels (**A**–**D**). (**E**) In addition, the proteins were collected and a Western blot was performed testing the membranes with anti-SIRT1. Histone 3 (H3) was used as a loading control. Graph (**F**) shows the mean ± SEM average of the integrated density of the SIRT1/H3 band ratio (*n* = 3). Graph (**G**) represents the mean ± SEM of the nuclear-integrated density of phospho-SIRT1 (*n* = 5 photos; more than 300 nuclei were counted in each image). Graph (**H**) shows the total SIRT1 and phospho-SIRT1 nuclear-integrated density of H_2_O_2_-treated groups. Statistics: One-way ANOVA followed by Tukey’s multiple comparisons test (**G**) and Šídák’s test for the comparison of group pairs (**H**). * *p* < 0.05; ** *p* < 0.01; *** *p* < 0.001. Scale bars represent 25 μM.

The phosphorylation of SIRT1 in serine 47 (Ser47) has been associated with the translocation of SIRT1 from the cytoplasm to the nucleus, increasing SIRT1 deacetylase activity at the nuclear level [64]. Therefore, we explored the nuclear expressions of SIRT1 and phospho-SIRT1 (Ser47) in 661W cells treated with 40 μM of PIC-OCT for 24 h. We observed that the nuclear fluorescent signal of SIRT1 and phospho-SIRT1 (Ser47) decreased in H_2_O_2_-treated cells (Figure 4B′–B), while the pretreatment with PIC-OCT prevented the decrease in nuclear expressions of SIRT1 and phospho-SIRT1 (Ser47) (Figure 4D′–D). When cells were not exposed to H_2_O_2,_, SIRT1 was expressed in the cytoplasm and nucleus in vehicle-treated cells as expected (Figure S3A,C), and 40 μM of PIC-OCT treatment increased the nuclear signal of SIRT1 (Figure S3B,D). The quantification of the nuclear signal of SIRT1 and phospho-SIRT1 increased significantly in the 40 μM of the PIC-OCT-treated group (Figure 4G,H). Additionally, WB analysis demonstrated that total SIRT1 protein expression decreased in cells exposed to H_2_O_2_, and PIC-OCT prevented the drop in SIRT1 protein levels (Figure 4E,F). These results were similar to those observed in the immunofluorescence studies (Figure 4A′–D).

### 3.4. PIC-OCT Blocked Intracellular ROS Production by Increasing Anti-Oxidant Gene Expression and Preserving the Mitochondrial Membrane Potential

**Figure 5:**
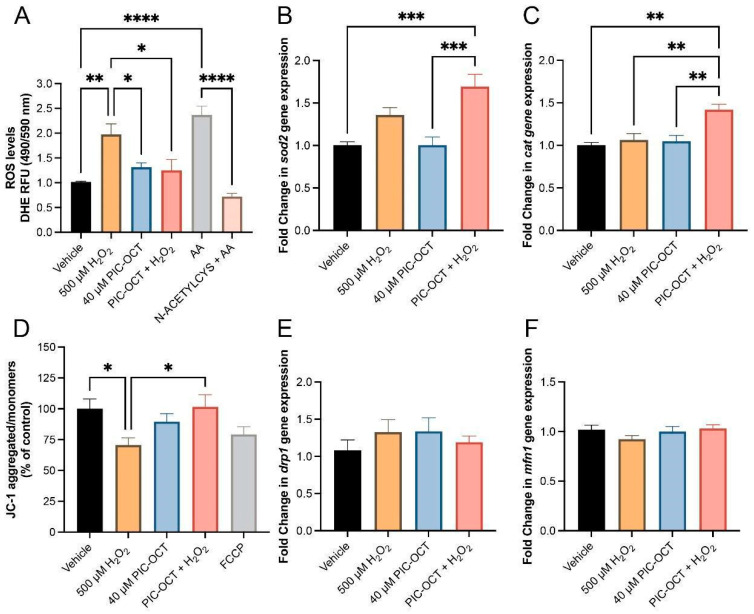
ROS levels, mitochondrial membrane potential, and gene expressions of anti-oxidant and mitochondrial dynamic genes. The 661W cells pretreated with 40 µM of PIC-OCT for 24 h were cultured and then exposed to 500 µM of H_2_O_2_ to mimic a severe acute model of oxidative stress. Intracellular ROS levels were detected by measuring the red fluorescence of DHE dye (**A**) (*n* = 3). ROS levels were significantly reduced in cells pretreated with PIC-OCT (**A**). Antimycin A (AA) and N-Acetyl cysteine (N-ACETYLCYS) were used as positive and negative controls, respectively. Effects of PIC-OCT on mRNA expression levels of antioxidant *sod2* (**B**) and *cat* (**C**) genes are shown in H_2_O_2_-treated 661W cells and pretreated with or without PIC-OCT for 24 h. The mRNA expression levels of *sod2* and *cat* for each sample were measured via real-time PCR; the fold changes in each gene are shown in the bar graph compared with the vehicle-treated group (*n* = 6). (**D**) The ΔΨm was evaluated via JC-1 staining (*n* = 3). PIC-OCT pretreatment preserved the ΔΨm in 661W cells exposed to H_2_O_2_. PIC-OCT slightly affected the expressions of the mitochondrial fission *drp1* (**E**) and fusion *mfn1* (**F**) genes. Statistics: One-way ANOVA followed by Tukey’s multiple comparisons test. * *p* < 0.05, ** *p* < 0.01, *** *p* < 0.001, **** *p* < 0.0001.

ROS are oxygen-containing reactive species such as hydrogen peroxide (H_2_O_2_), superoxide anion (O_2_^•−^), and hydroxyl radical (^•^OH). ROS are produced under normal conditions as products of oxygen metabolism and have a fundamental role in different cellular signaling pathways. However, when ROS accumulate in excess, they overwhelm the cellular defense mechanisms that control their homeostasis, leading to lipid and protein oxidation, DNA damage, and cell membrane and organelle dysfunction. Given that PIC-OCT protects 661W cells against H_2_O_2_ exposure (Figure 2 and Figure S2), the intracellular ROS levels were evaluated in H_2_O_2_- and PIC-OCT-treated cells, measuring the DHE dye’s red fluorescence. As expected, 661W cells treated with 500 µM of H_2_O_2_ showed a statistically significant increase in ROS levels (Figure 5A; Vehicle = 1.016 RFU), almost doubling the levels observed in the vehicle-treated group (Figure 5A; H_2_O_2_ = 1.973 RFU). Instead, pretreatment with PIC-OCT blocked the increase in ROS levels produced by the H_2_O_2_ exposure (Figure 5A). Antimycin A (AA) and N-Acetyl cysteine + AA (N-ACETYL-CYS) were used as positive and negative controls for ROS production, respectively (Figure 5A).

Moreover, it has been reported that SIRT1 overexpression increased nuclear factor E2-related factor 2 (Nrf2) deacetylation and its activity in a myocardial ischemia/reperfusion injury mouse model [65]. Nrf2 regulates the expression of antioxidant proteins and protects cells against ROS toxicity. Therefore, *sod2* (superoxide dismutase) and *cat* (catalase) gene expressions were evaluated in 661W cells treated with PIC-OCT (Figure 5B,C). Pretreatment with PIC-OCT significantly increased the expressions of *sod2* and *cat* when 661W cells were exposed to oxidative stress produced by H_2_O_2_ treatment. Since oxidative stress impacts mitochondrial function and morphology, the ΔΨm was evaluated through the formation of aggregates or monomers of JC-1 dye. H_2_O_2_ exposure produced a significant drop in ΔΨm, as expected. At the same time, PIC-OCT maintained membrane potentials that were similar to those observed in the mitochondria of vehicle-treated cells (Figure 5D). The expressions of mitochondrial fission *drp1* (Figure 5E) and fusion *mnf1* (Figure 5F) genes were also evaluated. Although an increase in the *drp1* gene expression was observed in H_2_O_2_-treated cells, and PIC-OCT treatment avoided the increase in *drp1* expression, these changes were not significant. On the other hand, the expression of *mnf1* slightly decreased in the H_2_O_2_-treated group. To evaluate the mitochondria morphology, the 661W cells were stained with MitoTracker (Figure S4; red). The cells treated with vehicle and PIC-OCT showed tubular mitochondria (Figure S4A–A′,B–B′) as expected, while in the H_2_O_2_-treated cells, the mitochondria showed fragmentation and swelling (Figure S4C–C′). The mitochondria in PIC-OCT pretreated cells exposed to H_2_O_2_ cells preserved their tubular structure without showing MitoTracker nuclear translocation (Figure S4D–D′).

### 3.5. PIC-OCT Treatment Decreases the Expression of Parthanatos Hallmarks in 661W Cells Exposed to H_2_O_2_

**Figure 6:**
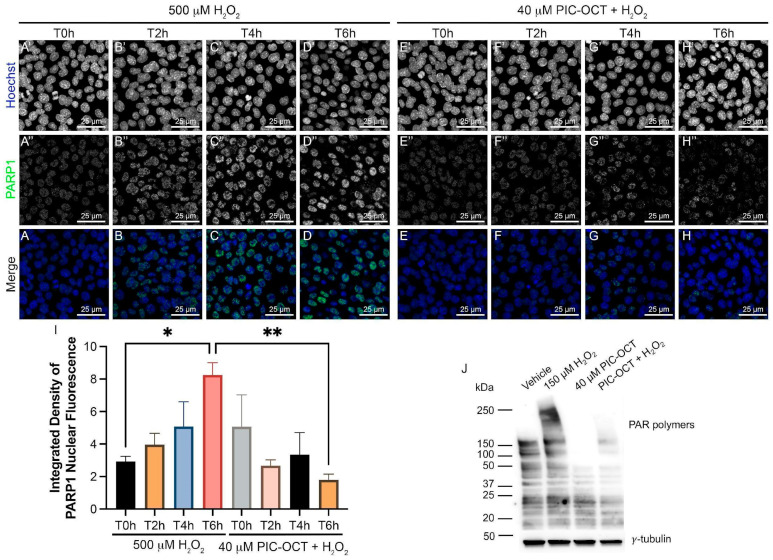
Expressions of PARP1 and PAR-polymers in H_2_O_2_- and PIC-OCT-treated 661W cells. The 661W cells were pretreated with 0.02% of DMSO (**A**–**A″**, **B**–**B″**, **C**–**C″**, and **D**–**D″**) or 40 µM of PIC-OCT (**E**–**E″**, **F**–**F″**, **G**–**G″**, and **H**–**H″**) for 24 h and then cells were exposed to 500 μM of H_2_O_2_ from 0 h (T0 h) up to 6 h (T6 h) (**A**–**D** and **E**–**H**). Then, the cells were fixed at T0 h, T2 h, T4 h, and T6 h and immunostained with anti-PARP1 antibodies (**A**–**H**; green). Hoechst dye was used to visualize the cell nuclei (blue). H_2_O_2_ exposure increases the expression of nuclear PARP1 (**B**–**D**), which was inhibited via PIC-OCT pretreatment (**F**–**H**). Scale bars represent 25 μM. The expression of PAR-polymers was evaluated via Western blot (**J**). The 661W cells pretreated with 40 µM of PIC-OCT for 6 h were cultured and then exposed to 150 µM of H_2_O_2_ for 18 h to mimic a chronic oxidative stress model. PIC-OCT decreases the expression of high molecular weight PAR-polymers in cells exposed to H_2_O_2_. Gamma-tubulin expression was used as a loading control (**J**). Graph (**I**) represents the mean ± SEM of the integrated density of PARP1 nuclear fluorescence (*n* = 5 photos; more than 300 nuclei were counted in each image). Statistics graph (**I**): One-way ANOVA followed Šídák’s test for the comparison of group pairs. * *p* < 0.05, ** *p* < 0.01.

To further confirm the protective effect of PIC-OCT, we examined the expressions of PARP1 and PAR-polymers in 661W cells exposed to H_2_O_2_. The cells were exposed to 500 µM of H_2_O_2_ alone (Figure 6A′–D) or with a 24 h pretreatment of 40 µM of PIC-OCT (Figure 6E′–H). The samples were taken at different time points (T0 h to T6 h). Then, the 661W cells were immunostained with anti-PARP1 antibodies (Figure 6A–H). H_2_O_2_ exposure clearly increased the nuclear expression of PARP1 (Figure 6A–D), which was more evident and statistically significant at T6 h (Figure 6I). On the contrary, PIC-OCT treatment almost wholly blocked the increase in PARP1 nuclear expression (Figure 6E–I). Moreover, PIC-OCT treatment decreased the synthesis of high molecular weight PAR-polymers observed after the chronic 150 µM of H_2_O_2_ exposure (Figure 6J). Therefore, pretreatment with PIC-OCT blocked two markers that typically initiate parthanatos, the recruitment of PARP1 at the nuclear level and the increase in PAR polymer synthesis.

**Figure 7:**
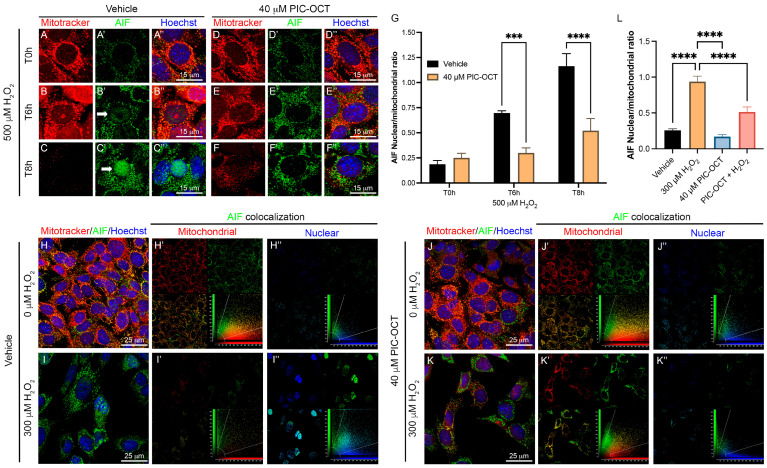
Mitochondrial and nuclear localization of the AIF signal in PIC-OCT-treated cells. The 661W cells were pretreated with a vehicle (0.02% DMSO) (**A**–**C″**,**H**–**I″**) or with 40 μM of PIC–OCT for 24 h (**D**–**F″**,**J**–**K″**) and then exposed to 500 μM of H_2_O_2_ for a further 6 and 8 h (**A**–**F″**) or 300 μM of H_2_O_2_ (**I**–**I″**,**K**–**K″**). The cells were stained with MitoTracker (**A**–**F**,**H**–**K**; red) and subsequently fixed with 4% PFA and immunostained with anti-AIF antibodies (**A′**–**F′**,**H**–**K**; green). Nuclei were visualized with Hoechst (**A″**–**F″**,**H**–**K**; blue). The co-localization of AIF (green) with the mitochondria (red) and nuclei (blue) are shown in different conditions (**A″**–**F″**,**H**–**K″**). The bars in graphs (**G**,**L**) represent the mean ± SEM of the ratio of nuclear/mitochondrial AIF colocalization. Statistics graph (**G**) (*n* = 3): Two-way ANOVA followed by Šídák’s test. Statistics graph (**L**) (*n* = 6): One-way ANOVA followed by Tukey’s test. *** *p* < 0.001, **** *p* < 0.0001. Scale bars represent 15 µm (**A**–**F″**) or 25 µm (**H**–**K″**).

AIF, a well-known downstream effector of PARP1 in parthanatos, is fundamental in the execution of H_2_O_2_-induced cell death via translocation to the nucleus. We investigated the downstream effect of PARP1 and the presence of AIF in the nucleus under oxidative stress. Hence, we examined the translocation role of AIF in 661W cells pretreated with 40 μM of PIC-OCT (Figure 7D–F″) in comparison with a second group pretreated with a vehicle of 0.02% DMSO (Figure 7A–C″). Both groups were exposed to 500 µM of H_2_O_2_ during 6 and 8 h (T0 h, T6 h, and T8 h). Staining was performed with MitoTracker (Figure 7A–C,D–F; red) and subsequently fixed with 4% PFA and then immunostained with anti-AIF antibodies (Figure 7A′–C′,D′–F′; green). The nuclei were stained with Hoechst (Figure 7A″–C″,D″–F″; blue). H_2_O_2_ exposure produced the translocation of the AIF signal from the mitochondria to the nucleus in vehicle-treated cells (Figure 7A–C″). This translocation was observed after 6 h of H_2_O_2_ exposure but was more evident at 8 h (Figure 7B″,C″; arrows). In contrast, cells pretreated with PIC-OCT did not show an AIF signal at the nuclear level despite having been treated with the same H_2_O_2_ dose (Figure 7E″–F″). In addition, H_2_O_2_ exposure produced swelling and fragmentation of mitochondria, and at T8 h almost all MitoTracker signals were lost in the vehicle-treated group. In contrast, this signal was preserved in the PIC-OCT-treated group (Figure 7C,F). Consistently, we quantified the ratio of nuclear/mitochondrial AIF colocalization in each condition and we observed a significant decrease in nuclear/mitochondrial AIF colocalization in 661W cells pretreated with PIC-OCT in comparison with the vehicle group at T6 h and T8 h (Figure 7G). We performed the same experiment using a lower dose of H_2_O_2_ (300 µM) (Figure 7I–I″,K–K″) for 8 h. Once more, PIC-OCT pretreatment preserved the colocalization of AIF with the MitoTracker signal (Figure 7K–K′) and avoided the AIF translocation to the nucleus (Figure 7K–K″). On the contrary, AIF colocalization was higher at the nuclear level than in the mitochondria in vehicle-treated cells exposed to H_2_O_2_. The quantification of the AIF nuclear/mitochondrial ratio in the images in Figure 7L corroborated the results.

### 3.6. PIC-OCT Treatment Inhibited the Formation of PAR-Polymers and Preserved Photoreceptors thus Improving Visual Behavior in rd10 Mice

**Figure 8:**
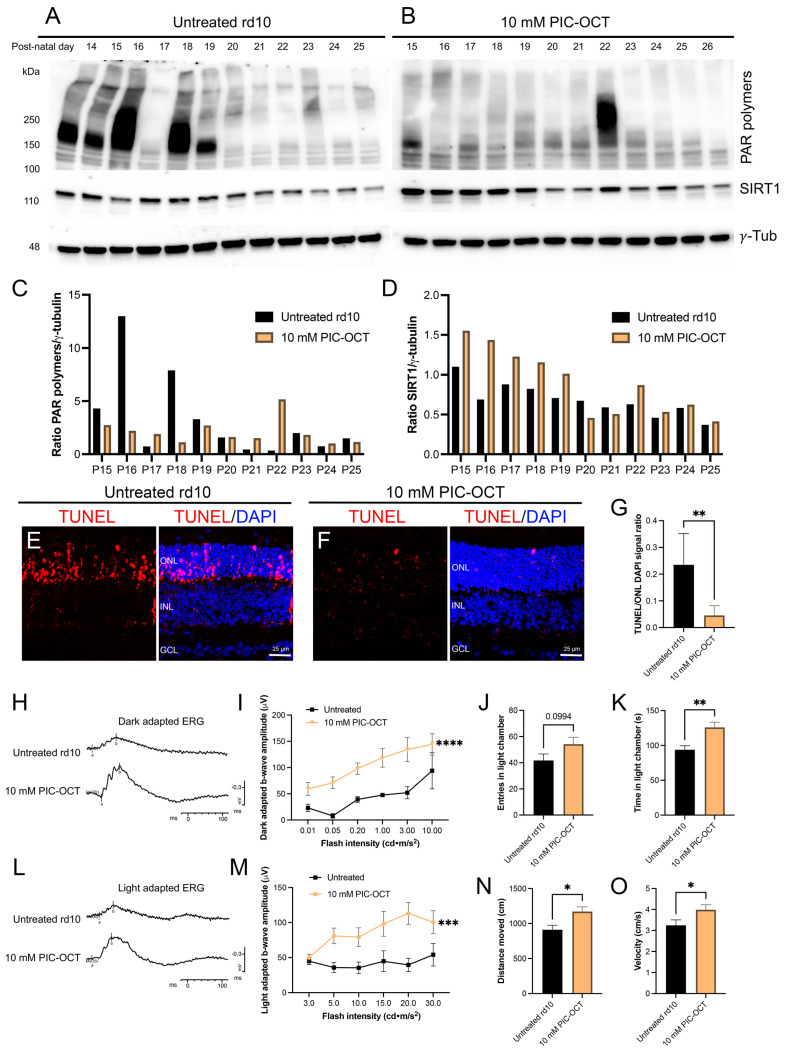
Formation of PAR-polymers, SIRT1 level, TUNEL staining, electroretinography evaluation, and light–dark box (LDB) test results in PIC-OCT-treated rd10 mice. Image (**A**) shows the neuroretina Western blot (WB) results proved with anti-PAR-polymers and anti-SIRT1 antibodies in untreated rd10 mice. Image (**B**) shows the results of the WB analysis of the rd10 mice treated with PIC-OCT. Gamma-tubulin (γ-Tub) expression was used as the loading control (**A**,**B**). Images (**C**,**D**) show the densitometry of the WB. The formation of high molecular weight PAR-polymers was inhibited with PIC-OCT treatment while the expression of SIRT1 increased (**A**,**B**). TUNEL signal (**E**, red) illustrates the number of dying photoreceptors in the outer nuclear layer (ONL) of untreated rd10 mice. In contrast, image (**F**, red) displays the TUNEL signal in rd10 mice treated with PIC-OCT at P18. These images additionally highlight distinct layers in the retinas of rd10 mice, where nuclei were stained with DAPI. Furthermore, (**G**) presents a graph quantifying the TUNEL signal in rd10 retinas. The analysis compares the TUNEL-positive signal with the ONL area across experimental groups (*n* = 7). Electroretinography (ERG) traces are shown from both untreated rd10 (*n* = 3) and PIC-OCT-treated mice (*n* = 6) in dark-adapted (**H**) and in light-adapted conditions (**L**). Graphs (**I**,**M**) represent the mean ± SEM of the b-wave amplitude of the rod (dark-adapted, **H**) and cone (light-adapted, **L**) responses. Different visual stimuli (candela (cd).s/m^2^) were used in each condition. PIC-OCT treatment significantly preserved the b-wave amplitude. The LDB test results are shown (**J**,**K**,**N**,**O**). Data are expressed as mean ± SEM of the number of entries in the light chamber (**J**), time spent in the light chamber (**K**), the distance moved, and velocity (**N**,**O**). PIC-OCT-treated mice showed higher time spent in the light chamber, distance moved, and velocity compared with the untreated group. INL: Inner nuclear layer; GCL: ganglion cell layer. Scale bars (**E**,**F**) represent 25 µm. Statistical analysis of graph (**D**–**F**): Two-way ANOVA *** *p* < 0.001, **** *p* < 0.001. Statistical analysis of graph (**G**–**J**): Student’s *t*-test (unpaired, two-tailed) for independent samples. * *p* < 0.05, ** *p* < 0.01.

We aimed to validate whether the formation of high molecular weight PAR-polymers was inhibited in rd10 PIC-OCT-treated mice (Figure 8A,C) as observed in 661W cells exposed to H_2_O_2_. The murine model of RP (rd10 mice) was injected intravitreally at postnatal day (P) P14 with 10 mM of PIC-OCT. The eyes were enucleated every other day, and protein extraction was performed with RIPA buffer, as described in Section 2. The formation of high molecular weight PAR-polymers was inhibited with PIC-OCT treatment from P15 to P19 when compared with untreated mice (Figure 8A,C). In addition, SIRT1 expression increases from P15 to P17 in the treated group (Figure 8B,D). 

Furthermore, we conducted TUNEL staining to evaluate cell death in the retinas of rd10 mice. The quantification of the TUNEL-positive signal is indicative of DNA fragmentation in dying cells, particularly in the ONL of P18 untreated rd10 mice (Figure 8E). The TUNEL assay demonstrated a significant reduction in the number of TUNEL-positive cells in the ONL of PIC-OCT-treated rd10 mice compared with untreated ones (*p* < 0.001, *t*-test) (Figure 8F,G).

In parallel, the retina’s electrical response to visual stimuli was evaluated at P28 via electroretinography (ERG). The rod response traces in dark-adapted mice (Figure 8H) and the cone response traces in light-adapted mice (Figure 8L) were evaluated. Quantifying the b-wave amplitude of the rod (Figure 8I) and cone (Figure 8M) responses showed that PIC-OCT treatment significantly preserved the b-wave amplitude at different visual stimulus intensities in both dark- and light-adapted conditions. The light–dark box (LDB) test was performed in dark-adapted mice for 8 h. PIC-OCT-treated mice showed higher time spent in the light chamber (Figure 8J), distance moved (Figure 8K), and velocity (Figure 8N) compared with the untreated group. Although the increase in the number of entries to the light chamber was not statistically significant (Figure 8O), this value, combined with the others, could reflect the improvement in light aversion in rd10 treated mice.

**Figure 9:**
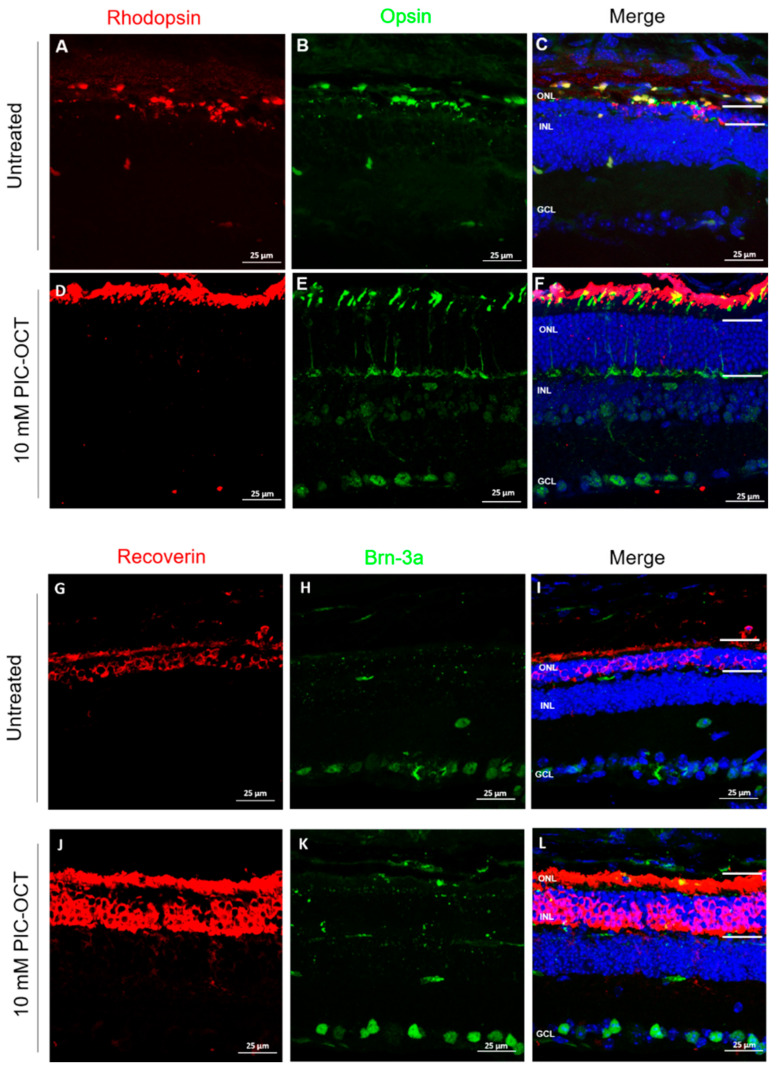
PIC-OCT prevented the degeneration of the retina architecture protecting photoreceptor cells in rd10 mice at postnatal day 28. Immunofluorescence of rd10 mouse retinas 15 days after subretinal injections (P28). The retinal sections were immunostained with anti-rhodopsin (**A**,**D**) and anti-opsin (**B**,**E**) antibodies. Immunolabelling of both types of photoreceptors (rods = red, cone = green) is shown. DAPI dye (blue) was used to stain the nuclei. The merge of both immunostaining can be observed in the right panel (**C–F**). Photoreceptors were also immunostained with recoverin (**G**–**J**). Retinal ganglion cells were labeled with anti-Brn-3a (**H**–**K**). DAPI dye (blue) was used to visualize the cell nuclei (blue) and the merge of the 3 channels it can be observed in the panels (**I**–**L**). ONL: Outer nuclear layer; INL: inner nuclear layer; and GCL: ganglion cell layer. Scale bars represent 25 µm.

Furthermore, the impact of PIC-OCT treatment on the retinal cells of rd10 mice was investigated 15 days after the IVT injections of PIC-OCT. Rhodopsin and opsin, which are light-sensitive proteins, are expressed in the outer segment (OS) of rod and cone cells, respectively [66]. Consequently, we utilized rhodopsin and opsin immunostaining to evaluate the expression and distribution of these molecular markers of photoreceptors (Figure 9A–F). In untreated rd10 mice, the rod OS exhibited poor rhodopsin staining, accompanied by a notable decrease in the ONL thickness (Figure 9A–C). Conversely, in PIC-OCT-treated rd10 mice, a substantial number of rod OS were noticeably stained, and the ONL thickness was preserved (Figure 9D–F). The administration of PIC-OCT resulted in the preservation of rhodopsin expression in rod OS (Figure 9D) and ONL thickness (Figure 9F). These findings align with those observed in nuclear staining, indicating a greater number of cell bodies in the ONL. Thus, PIC-OCT treatment effectively decelerated the degeneration of rd10 rods. Moreover, cones undergo degeneration in rd10 mice, albeit at a later stage than rods [67]. Subsequently, we evaluated the protective impact of PIC-OCT on cone structure via opsin immunostaining. The expression of opsin markedly decreased in untreated rd10 mice compared with 10 mM PIC-OCT-treated rd10 mice (Figure 9B,E), suggesting that PIC-OCT treatment also impeded the degeneration of rd10 cones. 

We also assessed the retinal degeneration in the rd10 mouse retina treated with PIC-OCT by examining recoverin immunoreactivity, which labels rod and cone photoreceptors (Figure 9G,J). We observed high expression of recoverin in rd10 mice treated with PIC-OCT in the ONL and OS compared with retinas treated with the vehicle (Figure 9G,J). Moreover, we explored the morphology and distribution of RGCs via immunostaining Brn-3a, a transcription factor highly specific for RGCs in the retina (Figure 9H,K). Regarding retina architecture during the degeneration process of rd10 mice, it has been described that RGCs do not show significant changes until nine months of age [68]. As expected, we found that PIC-OCT did not induce significant changes in RGCs at P28 compared with the untreated group (Figure 9H,K).

## 4. Discussion

Numerous studies have highlighted the anti-inflammatory and antioxidant properties of polyphenol stilbenes, such as RSV, and their beneficial effects in metabolic, cardiovascular, and neurodegenerative diseases, including retinal degenerative conditions [21,22,23,24,25,26,27,46,47,69]. Despite the potential therapeutic effect of natural polyphenols, their exact mechanisms of action still need to be well understood. Furthermore, limited oral bioavailability, low water solubility, and rapid metabolism have limited the use of these beneficial compounds to treat different human pathologies [43,69]. Therefore, synthesizing new molecules derived from polyphenols with better pharmacokinetic properties is critical to ensure their effectiveness and clinical translation to the clinic. This study supports the protective effects of PIC-OCT treatment on photoreceptor degeneration. PIC-OCT is an innovative molecule designed to improve the RSV pharmacokinetics that may serve as a promising candidate for clinical trials in RP.

It has been reported that enzymatic oxidation of polyphenols can be retarded by glycosylation of these molecules, extending their half-life [70]. PIC is a natural glycoside derivative of RSV that has shown higher ROS scavenging activity than RSV in vitro. PIC may undergo enzymatic hydrolysis in the colon or enterocytes, leading to trans-RSV formation, offering an alternative for soluble RSV administration [71]. The new molecule PIC-OCT includes PIC in its structural composition to enhance the therapeutic efficacy of RSV. Although PIC might offer an opportunity for oral administration of RSV, it has been described that PIC has limited cellular uptake [71,72]. Medium-chain fatty acids (MCFAs), including OCT, have been used in formulation to improve the druggability of certain small molecules. PIC-OCT also includes an octanoyl chain (C8), which might enhance cell uptake of this molecule. In addition, it has been described that OCT also modulates inflammation and improves neuronal development, which may suggest a multifaceted pharmacological effect of PIC-OCT beyond being just a prodrug [24,46].

Herein, in the context of the ADME (administration, distribution, metabolism, and excretion) in silico prediction studies, PIC-OCT displayed several indispensable attributes to be a drug-like candidate. The discernment furnished by the bioavailability radars conspicuously underscores an elevated liposolubility inherent to PIC-OCT, thereby making it discernibly better than the profiles of both RSV and PIC. Yet guided by the tenets set forth by Lipinski’s principles [73], it is prudent to acknowledge that the flexibility and polarity of PIC-OCT might not be optimally aligned for oral administration. Mindful of this pivotal consideration, our laboratory remains steadfastly dedicated to designing innovative pharmaceutical formulations for PIC-OCT for intraocular or topical administration through ocular instillation [44].

The beneficial impact of RSV on a retinal degeneration model has been previously documented in both in vitro and in vivo models [21,22,27,47]. While the protective effect of RSV against photoreceptor death has been established in models involving glucose deprivation or light exposure in 611W cells [21], studies led by Juan Carlos Morales’ research group reported an enhanced protective response of PIC-OCT compared with RSV in a neurodegenerative animal model for the first time [24]. In the context of prior investigations involving PIC-OCT, it was observed that this compound facilitated the delivery of both RSV and OCT. Notably, these substances remained detectable in cells 72 h post-treatment in various cancer cell lines, which was a contrast to the rapid metabolism observed with RSV treatment [74]. This earlier study underscores the improved delivery properties of PIC-OCT. Furthermore, within our research group, a preliminary screening revealed that PIC-OCT exhibited superior protective qualities for the retina in rd10 mice [47] compared with RSV and JC-19 [22]. Specifically, we found in rodent models, in rd10 mice, that PIC-OCT administered subretinally delayed photoreceptor degeneration to a greater extent than RSV or JC-19 [47]. Moreover, in another study, orally administered PIC-OCT reduced colon inflammation in the murine dextran sulfate sodium (DSS) model [46], showing promising results as a potent anti-inflammatory drug.

Presently, we are delving into the role of PIC-OCT in both in vitro and in vivo models of retinal degeneration. This exploration aims to advance our understanding of the molecular mechanisms associated with PIC-OCT and represents the initial steps toward a prospective clinical trial. This study found that PIC-OCT increased SIRT1 activity. SIRT1, one of seven known sirtuins, is located in the cytoplasm and nucleus, and it has been involved in response to molecular damage and metabolic imbalance triggered by the production of ROS [31]. In addition, activation of SIRT1 is involved in stress response, autophagy, and apoptosis and can modulate inflammation and facilitate the degradation of misfolded proteins through the deacetylation of numerous transcription factors [14,75]. Liu et al. demonstrated that the administration of RSV protects 661W cells by activating SIRT1 [21]. Hence, SIRT1 activation might have a promising therapeutic role for retinal degenerative diseases. RSV is known for increasing SIRT1 activity and protecting against oxidative damage in various neurodegenerative disease models [23], but whether there is a direct interaction between SIRT1 and RSV is still controversial. Herein, we described that PIC-OCT treatment, besides increasing SIRT1 activity, also upregulated the nuclear expressions of SIRT1 and phospho-SIRT1 (Ser47) in 661W cells. It has been reported that phosphorylation of SIRT1 by c-Jun N-terminal kinase 1 (JNK1) increased its nuclear localization and enzymatic activity [64]. In addition, inhibition of mitogen-activated protein kinase (MAPK) has shown a regulatory role in SIRT1 activity [76], and protein target and network analysis predict the interaction of RSV with several kinases, including MAPK3 and MAPK4 [77]. Therefore, these results suggest that PIC-OCT not only activates SIRT1 but could also favor its phosphorylation and nuclear translocation. Further experiments are required to confirm the interaction of PIC-OCT with members of the MAPK family of kinases. In this line, knockout experiments are required to better understand the molecular mechanism underlying the protective effects of this molecule. In this context, we could advance in the identification of specific targets for new pharmacological strategies for RP.

This study unraveled the molecular mechanism behind PIC-OCT’s protective impact on cell viability during cell death. Our focus was on the parthanatos signaling pathway, known for its significant involvement in photoreceptor degeneration-induced cell demise due to oxidative stress by PARP overactivation. As mentioned above, PARP inhibitors have been considered a feasible alternative in RP pharmacological management. In the realm of in vitro studies with PARP inhibitors, Olaparib has shown promise in rescuing rd1 photoreceptors within short-term retinal explant cultures with a dose-dependent relationship. Extending the treatment to P17 demonstrated increased photoreceptor rows and decreased TUNEL-positive cells within the Olaparib-treated groups. Further experiments extending treatment to P24 did not yield significant effects on photoreceptor survival. This indicates that while Olaparib delayed photoreceptor degeneration, it could not entirely prevent it long term [15]. In the 661W cells, Olaparib failed to prevent TNFα-induced cell death at concentrations of 1.5, 5, and 10 μM when administered 2 h before stimulation with tunicamycin (10 μg/mL) or TNFα (100 ng/mL) [78]. Although Olaparib has shown neuroprotective effects in in vivo studies, it can cause adverse effects in cancer-treated patients during long-term use. These include hematological issues (anemia, thrombocytopenia, and neutropenia) mainly in the first three months, sometimes necessitating dose adjustments. Non-hematological side effects (digestive tract toxicity, hepatotoxicity) typically appear within the first 4–8 weeks but are often manageable without reducing the dosage. Common side effects include nausea, vomiting, and fatigue. Close monitoring is essential during PARP inhibitor therapy [19]. Therefore, repositioning PARP1 inhibitors for their systemic chronic use in RP could be challenging, opening the possibility to other less toxic inhibitors that could be administered topically.

Regarding the photoreceptor degeneration pathway, prior research has highlighted that severe DNA damage prompts an excessive activation of PARP1. This fact drives the dissipation of the ΔΨm and the migration of AIF from mitochondria to the nucleus, culminating in caspase-independent cell death. Moreover, AIF’s capacity to induce cytochrome c release from purified mitochondria suggests that AIF expedites membrane permeabilization upon liberation from mitochondria, fostering a constructive feedforward loop [20,21,79,80]. The outcomes showcased a noteworthy decline in nuclear expression of PARP1 protein levels through PIC-OCT, resulting in decreased PAR polymer accumulation and subsequent reduction in mitochondrial AIF release. These findings align with our presumption of PIC-OCT triggering neuroprotection for photoreceptors by inhibiting parthanatos. Previous studies have documented that RSV significantly suppresses the light-exposure-induced upregulation of both PARP1 and AIF protein expressions in 661W cells, corroborating our results [21]. Beyond the protective role of parthanatos inhibition in photoreceptor degeneration, the beneficial effect of inhibiting this cell death pathway by natural polyphenols has also been described in other neurodegenerative disease models such as Parkinsonism induced by 1-methyl-4-phenylpyridinium ion (MPP+) [79]. This fact provides compelling evidence supporting the notion that neuronal loss can be prevented by inhibiting the PARP-dependent cell death pathway. 

The parthanatos cell death molecular mechanism usually underlies an imbalance between pro-oxidant and antioxidant factors in cells triggered by severe oxidative stress. Regulating the expression or activity of the antioxidant enzymes is a target to maintain the redox balance that may avoid DNA damage. Genes encoding phase II metabolizing enzymes and oxidative stress-related genes such as *CAT* and *SOD2* have been described to be reduced by H_2_O_2_ compared with the control in retinal pigment epithelial cells (ARPE-19) [81]. This study demonstrated that pre-incubation with PIC-OCT increased *cat* and *sod2* gene expressions in cells injured with H_2_O_2_ compared with the vehicle-treated cells, thus suggesting that PIC-OCT can struggle with parthanatos by increasing antioxidant enzymes. It has been described that high doses of H_2_O_2_ (750 μM) in ARPE-19 cells induce severe oxidative stress and thus prevent Nrf2 mRNA expression and decrease the activity of this master regulator of antioxidant enzyme response; however, 200 μM of H_2_O_2_ can induce moderate oxidative stress and cause Nrf2 activation, increasing mRNA and protein expressions of antioxidant genes [82,83,84]. Herein, we observed that 500 µM of H_2_O_2_ alone during 6 h did not decrease *cat* and *sod2* mRNA expressions compared with untreated cells. This suggests that these doses maintain the antioxidant enzyme response in 661W cells, and PIC-OCT pretreatment increased it. Although we do not know how PIC-OCT can increase the expression of antioxidant genes, we believe that its action is mediated through SIRT1 since it has been described that SIRTI can deacetylate FOXO3a and Nrf2 and activate their transcriptional activity to upregulate downstream protective genes [65,85].

Since PARP1 and SIRT1 have a role in DNA repair, they have been proposed as a point of convergence for this process [14,35]. PARP1 uses NAD^+^ as a substrate for its ADP-ribosylation activity as part of the DNA repair mechanism and as one of the initial steps of the parthanatos process. SIRT1, like PARP1, shares the same NAD^+^ cofactor to carry out enzymatic activity [14]. Therefore, SIRT1 could influence PARP1 activity by decreasing the NAD^+^ pool and vice versa [13,14]. We found that the treatment with PIC-OCT increased the ratio of NAD^+^/NADH compared with 661W untreated cells, indicating that our drug improves the redox balance in vitro; therefore, in our case, the activation of SIRT1 by PIC-OCT would not seem to decrease the NAD^+^ pull to reduce the PARP1 activity. We do not know why PIC-OCT preserves NAD^+^ levels, but we assume that it could be related to the chemical composition of the molecule. It has been reported that ketones can increase the redox NAD^+^/NADH ratio in the human brain [86]. OCT and other MCFA, such as decanoic acid (C10), are ketone precursors. Therefore, the presence of OCT in the PIC-OCT molecule could also have a role in the positive redox NAD^+^/NADH ratio observed in PIC-OCT-treated cells. NAD^+^ homeostasis plays an essential role in retinal metabolism and cell survival; thus, NAD^+^ intermediates may have a potentially beneficial effect in treating retinal degeneration [32]. A neuroprotective effect of nicotinamide mononucleotide (NMN), an NAD^+^ precursor, has been previously reported in a model of photoreceptor degeneration [87]. In a retinal detachment model, NMN treatment has shown an increment in retinal NAD^+^ levels and upregulated SIRT1 and heme oxygenase-1 (HO-1). Moreover, in vitro studies revealed a neuroprotective effect of NMN in ROS-induced 661W cell death, probably through the SIRT1/HO-1 signaling pathway. In this context, Chen et al. showed that NMN treatment in vivo normalized excessive ROS production and increased the antioxidant responses. They found that the protective effects of NMN on 661W cells under oxidative stress are partially SIRT1-dependent because of the role of other sirtuin members [88,89,90]. Herein, we highlight the multi-target attribute of PIC-OCT by serving as a ROS scavenger through the activation of SIRT1 with the consequent inhibition of PARP1 and coupled with the maintenance of NAD^+^ homeostasis.

Mitochondrial dysfunction plays an important role in degenerative retinal diseases such as RP and age-related macular degeneration (AMD) [81,91,92,93]. Notably, within a recent investigation focused on in vitro retinal damage induced by H_2_O_2_, mirroring emulating the oxidative damage model employed in our present study, it was discovered that maintaining mitochondrial proteostasis provides neuroprotection against oxidative injury in retinal degenerative diseases (RDDs) [94]. Interestingly, the study revealed that elevated concentrations of H_2_O_2_ did not lead to a significant increase in cell death. Instead, the activation of both mitophagy and the mitochondrial unfolded protein response (mtUPR) demonstrated a positive correlation with enhanced cell viability, concurrently diminishing apoptosis and oxidative damage levels in 661W cells subjected to H_2_O_2_ treatment [94]. mtUPRs encompass various compensatory processes associated with proteostasis and antioxidant mechanisms. Their activation, triggered by an overcompensation response to mild intracellular stress, plays a crucial role in promoting cell homeostasis. This activation has been shown to enhance lifespan and ameliorate disease-related alterations in biological models of mitochondrial dysfunction, including age-related diseases, cardiopathies, metabolic disorders, and primary mitochondrial diseases [95]. Moreover, the protective impact of both mitophagy and mtUPR on mitochondria was evident in the augmentation of the ΔΨm and the preservation of mitochondrial mass. Conversely, hindering mitophagy reversed the favorable outcomes associated with mitophagy in 661W cells treated with H_2_O_2_. Collectively, these findings propose that targeting the mitophagy and mtUPR pathways could offer novel therapeutic avenues to impede the advancement of retinal degenerative diseases (RDDs) by fortifying mitochondrial proteostasis [94]. Expanding on the noteworthy outcomes previously disclosed, our current focus involves investigating the influence of PIC-OCT on autophagy and proteasome activity. Following this amazing result previously reported, we plan to study the effect of PIC-OCT on autophagy and proteasome activity.

Precisely, mitochondria exposed to oxidative damage have been shown to exhibit severe morphological changes promoting mitochondrial dysfunction [96,97]. In our study, compared with the normal elongated, filamentous mitochondria, cells exposed to oxidative damage had more oval and round mitochondria. Moreover, we found that the mitochondria under H_2_O_2_ exposure were depolarized compared with the control cells. However, treatment with PIC-OCT significantly attenuated changes in ΔΨm, as indicated by a significant increase in the aggregated/monomer ratio compared with the H_2_O_2_ group. 

Additionally, we explored the effect of PIC-OCT on regulating the mitochondrial dynamic process. We selected key mitochondrial dynamic players (*MFN1* and *DRP1)* to investigate the effects of PIC-OCT on mitochondrial function under oxidative damage. We did not appreciate any important change in the mRNA expressions of *drp1* and *mfn1*. Still, we observed a remarkable swelling in MitoTracker-stained cells treated with H_2_O_2_, but cells pretreated with PIC-OCT maintained elongated morphology even with oxidative damage exposure. These findings were appreciated in a particular context of chronic oxidative damage. This result aligns with the rescue in the ΔΨm induced by PIC-OCT in 661W injured cells. Similar results of maintaining mitochondrial function and morphology have been reported in RPE cells pretreated with an antioxidant drug, triphenylphosphonium (TPP)-Niacin, and then exposed to H_2_O_2_ [98], as well as in 661W cells treated with RSV and injured either by glucose deprivation or light exposure [21]. Here, we highlight the effect of PIC-OCT on mitochondrial dynamic protection as a strategy that can increase the beneficial effect against photoreceptor degeneration.

Regarding the in vivo experiments, previous reports have shown that the nuclear SIRT1 expression decreased in the outer nuclear layer (ONL) of rd10 mouse retinas at P15, and this unusual expression pattern was correlated with the onset of photoreceptor degeneration [99]. The findings presented in this study revealed an elevated expression of retinal PAR-polymers during the third postnatal week of rd10 mice. PIC-OCT intravitreally injected mice showed higher Sirt1 retinal expression three days after injections (P15–P17). It was remarkable that low levels of PAR-polymers were observed in the same retinas at the same period, and such PAR-polymer expression was observed only in one retina days after. Additionally, we detected a significant decrease in TUNEL signals in the ONL of mouse retinas treated with PIC-OCT compared with untreated rd10 mice. Previous histological evaluation of rd10 mouse retinas at P18 has demonstrated a high number of TUNEL-positive cells in the ONL as well as high numbers of PARP- and PAR-stained photoreceptors but without detection of mitochondrial cytochrome c release or caspase-9 activation. These interesting findings described by Arango-Gonzalez et al. [9] support our results that highlighted the activation of a caspase-independent cell death mechanism in rd10 mouse retinas and point out the promising therapeutic target of modulating the balance of the SIRT1/PARP1 axis in RP.

Moreover, in untreated and PIC-OCT-treated mice, the electrical responses of the retina to visual stimuli and the aversion to light were evaluated through ERG and the LDB test. The best ERG traces at different flash intensities of PIC-OCT-injected mice were observed in both dark- and light-adapted mice, suggesting that either rod or cone photoreceptors, as well as other neuronal components within the retinas, were preserved after PIC-OCT treatment [22,47]. Under dark-adapted conditions, ERG b-wave also provides indirect information about retinal ganglion cell (RGC) activity [100]. In this context, further experiments, such as pattern electroretinogram (PERG) recording, are required to understand better whether PIC-OCT also exerts a therapeutic effect on RGC activity.

Finally, we performed a behavioral test to evaluate the improvement in photophobia in the treated mice. Photophobia is a symptom frequently observed in RP and can significantly affect the patient’s quality of life [101]. To evaluate the anxiety-like behavior produced by the innate aversion of nocturnal rodents to brightly illuminated areas, we used the LDB test [102]. This test consists of two compartments connected by an opening. Only one of the compartments is illuminated, while the other is not [103]. Under normal conditions, wild-type mice tend to stay longer in the dark area since their exploratory behavior toward a new environment is suppressed by the negative stimulus produced by the illuminated area. In the case of rd10 mice, it has been described that the time they remained in the dark chamber was longer, and the rejection of the illuminated compartment increased when light intensity was higher [104]. This aversion to the light of rd10 has been explained by the persistence of photosensitive elements within the degenerative retinas of rd10 mice [104]. PIC-OCT-treated mice spent more time in the illuminated chamber than untreated ones, who showed rejection of the illuminated compartment, with less access and time spent in the light chamber. In addition, the exploratory behavior represented by the velocity and distance were also higher in the rd10-treated mice. These results indicate that PIC-OCT could reduce photosensitivity and anxiety in rd10 mice. Although it has been shown that RSV chronically administered increases the time spent in the open arms during the elevated plus maze test, effectively mitigating anxiety-like behaviors induced by maternal separation in mice [105], we assume that our effect could be more related to the preservation of photoreceptors and intra-retinal neuronal circuits since we have used a single intravitreal injection and not a chronic and systemic administration, as is usually used with anxiolytic drugs.

In the context of retinal architecture, our investigation has substantiated that PIC-OCT effectively mitigates degeneration in rods and cones. However, the specific posology employed in our current study did not yield significant alterations in RGCs. This outcome is consistent with the known pathophysiology of retinal degeneration (RD) in rd10 mice. In rd10 mice, RD onset occurs typically around P18–20, marked by the progressive demise of rod photoreceptors. The loss of rod photoreceptors extends until P45–70. Although the somas of cone photoreceptors persist until P270–285, degeneration in the outer and inner segments of cone photoreceptors begins before P70. Notably, a 20% reduction in rod bipolar cells emerges between P45 and P105, and a 39% decrease in horizontal cells is observed between P45 and P270. Intriguingly, RGCs do not exhibit significant changes until the mice reach nine months of postnatal age [68,106]. Exploring the enduring impact of PIC-OCT on rd10 mice through an extended treatment could offer valuable insights, providing a robust confirmation of its effects on RGCs.

For future work, we propose to test our molecule in 3D models of retinal organoids derived from iPSCs obtained from RP patients to verify the effectiveness of PIC-OCT in different RP models independent of the mutations that cause the disease. These experiments will enhance our understanding of the pan-therapeutic protective mechanisms of PIC-OCT within more representative RP human disease models. Moreover, in the context of personalized medicine, transcriptomics studies should be performed to better understand the molecular mechanisms that are underlying the protective effect of PIC-OCT on IRDs, working these different RP mutations [107]. Further investigations into the toxicity and biodistribution of PIC-OCT are indispensable to establish the safety and ADME profile of this new molecule for potential use in a clinical trial.

## 5. Conclusions

**Figure 10:**
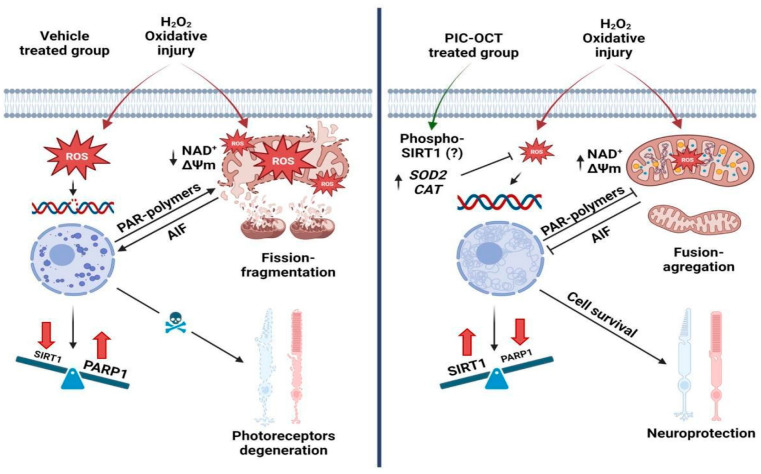
Depicting the effect of PIC-OCT in photoreceptor precursors upon oxidative damage. The 661W cells were pretreated with the vehicle or PIC-OCT and then exposed to H_2_O_2_ to induce oxidative damage. PIC-OCT increases the expression of phospho-SIRT1 at the nuclear level, probably through direct or indirect activation of an unknown kinase. Moreover, we observed an increased NAD^+^/NADH ratio in the PIC-OCT group in highly NAD^+^-consuming cells due to PARP1 overactivation. Our results show a decrease in ROS in cells pretreated with PIC-OCT exposed to oxidative damage, showing a potential role as a ROS scavenger increasing *sod2* and *cat* expressions. PIC-OCT decreases PARP1 expression and suppresses PAR-polymer levels and nuclear translocation of AIF, which then decreases parthanatos activation. PIC-OCT shifts the balance between PARP1 and SIRT1, increasing the antioxidative response. PIC-OCT maintains mitochondrial potential membrane and mitochondrial morphology even under oxidative conditions. These findings suggest that PIC-OCT protects retinal cells in an in vitro oxidative stress model, showing significant antioxidant effects delaying degeneration and cell death through the modulation of SIRT1/PARP1 balance.

Our study states that PIC-OCT can protect against oxidative stress in photoreceptor-like cells [108] exposed to H_2_O_2_ (Figure 10) and in the rd10 mouse model through the modulation of the SIRT1/PARP1 axis inhibiting the parthanatos pathway. This hypothesis was confirmed in vitro by inhibiting PARP1 and PAR-polymer expressions and decreasing the AIF nuclear translocation. In rd10 mice, PAR-polymer expression was also inhibited, preserving retinal function. All these findings support using PIC-OCT in future regulatory preclinical studies.

## Figures and Tables

**Table 1:** List of gene expression assays.

TaqMan^®^ Assay	Function	Gene Symbol
Assay ID: Mm00679214_g1	Mitochondrial fission	*Dnm1/Drp1*
Assay ID: Mm00612599_m1	Mitochondrial fusion	*Mfn1*
Assay ID: Mm01313000_m1	Superoxide dismutase 2, mitochondrial	*Sod2*
Assay ID: Mm00437992_m1	Peroxidase	*Cat*
Assay ID: Mm99999915_g1	Dehydrogenase housekeeping	*Gapdh*

Primers used for gene expression analyses in real-time qPCR assays.

## Data Availability

Data is contained within the article and supplementary material.

## Supplementary Materials

The following supporting information can be downloaded at: https://www.mdpi.com/article/10.3390/antiox13020201/s1. Figure S1: Cell viability of 661W pretreated with PIC and exposed to H_2_O_2_. Figure S2: Cell viability of 661W cells pretreated with PIC-OCT and chronically exposed to H_2_O_2_. Figure S3: Nuclear and cytoplasmic SIRT1 expression in PIC-OCT-treated cells. Figure S4: Mitochondrial morphology in PIC-OCT-treated cells.
