# Supplementary material for: Piceid Octanoate Protects Retinal Cells against Oxidative Damage by Regulating the Sirtuin 1/Poly-ADP-Ribose Polymerase 1 Axis In Vitro and in rd10 Mice
Source: Antioxidants (Basel). 2024 Feb 4;13(2):201. doi: 10.3390/antiox13020201 (PMC10886367; doi:10.3390/antiox13020201)
Supplement: Supplementary file 1 [file antioxidants-13-00201-s001.zip › antioxidants-2833855-supplementary.pdf]

Supplementary Figures.

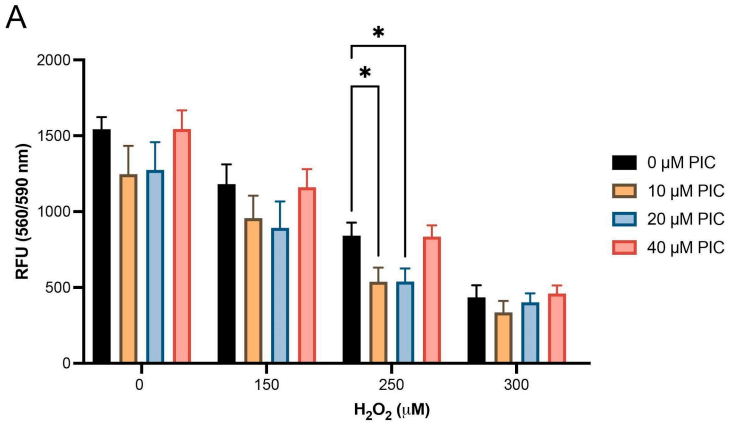

Supplementary Figure S1. *Cell viability of 661W pretreated with PIC and exposed to H_2O_2.* PIC treatment did not protect 661W cells against oxidative stress. Cells were grown in 96-well plates and pretreated with PIC (10-40 μM) for 24 h. Then, 661W cells were exposed to H_2O_2 (150-300 μM) for 6h. 0.02% DMSO was used as the vehicle. Finally, cell viability was measured with the CellTiter-Blue assay. Graph (A) shows the cell viability of 661W cells treated with different doses of PIC and exposed to increasing doses of H_2O_2. PIC did not show a cytoprotective effect against H_2O_2 exposure; even cell viability was significantly lower in 10-20 μM PIC-treated cells and exposed to 250 μM H_2O_2. The graphs´ bars represent the mean ± SEM of relative fluorescence units (RFU) (560/590 nm) (n= 3). Statistics: Two-way ANOVA followed by Dunnett's test to compare different groups with the control (0 μM PIC). * $p<0.05$

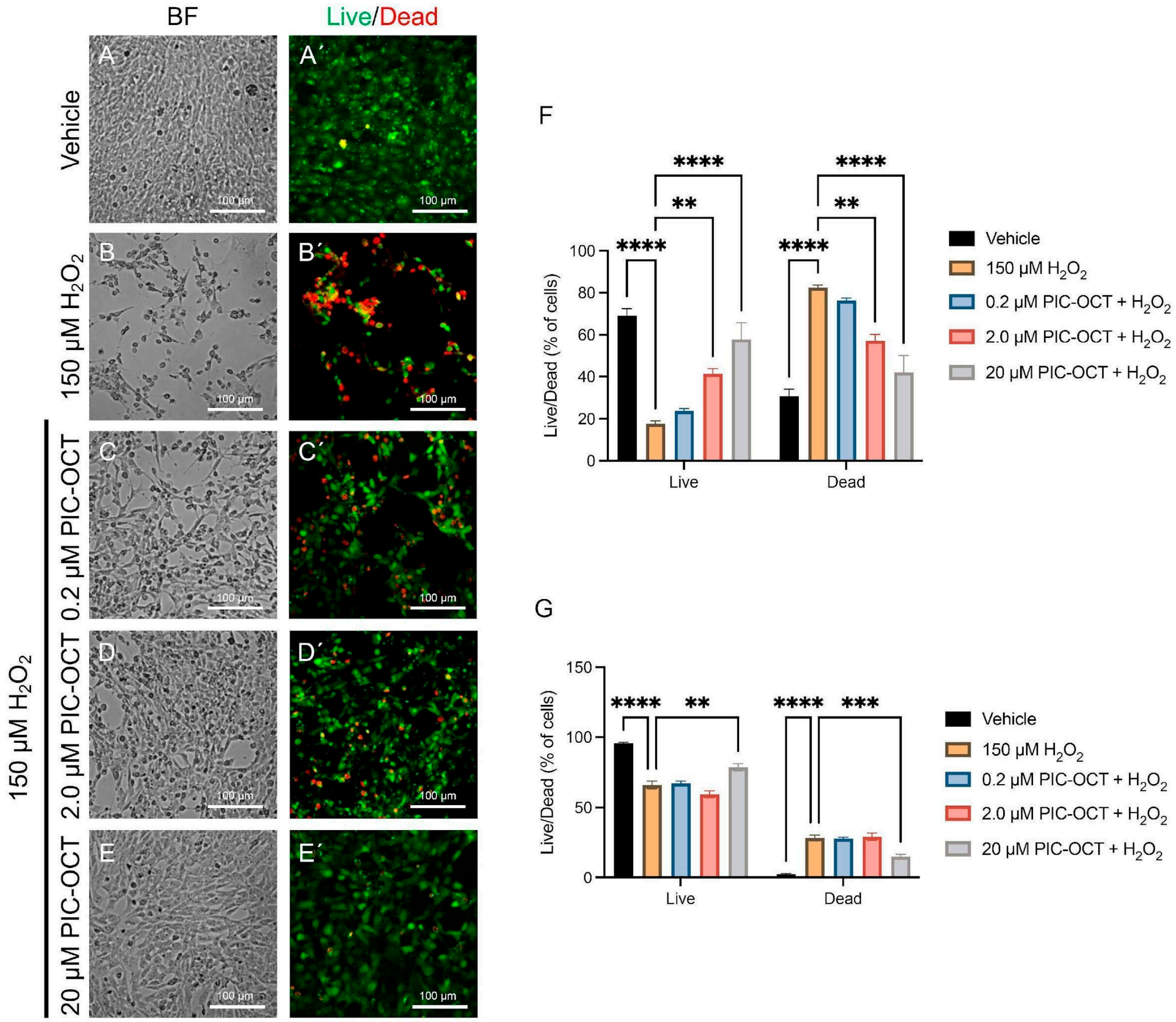

Supplementary Figure S2. *Cell viability of 661W cells pretreated with PIC-OCT and chronically exposed to H_2O_2.* PIC-OCT protects 661W cells against chronic H_2O_2 exposure. 661W cells were treated with 0.2, 2 or 20 µM of PIC-OCT for 12 h and then cells were exposed to 150 µM H_2O_2 for 24 h. Then, the cell viability/cytotoxicity was evaluated by bright-field (BF) images (A-E) and the Live/Dead staining kit (A'-E'). The graphs represent the mean ± SEM of cell viability measured by a cell counter (F) or cytometry (G) (n= 3). Scale bars represent 100 µm. Statistics: Two-way ANOVA followed by Dunnett's test. ***p*<0.01, ****p*<0.001, *****p*<0.0001.

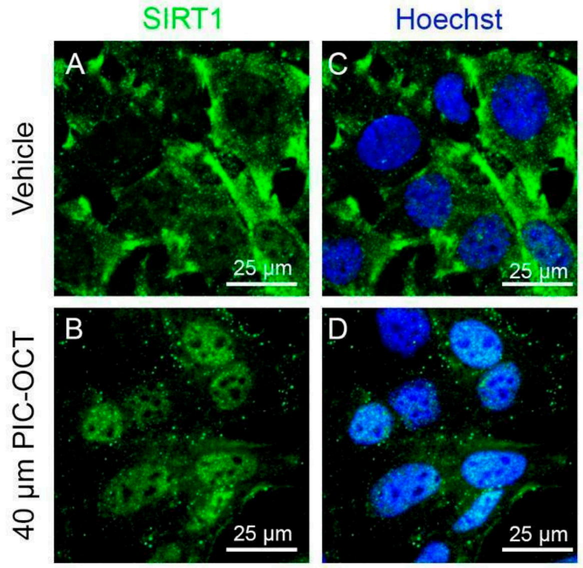

Supplementary Figure S3. *Nuclear and cytoplasmic SIRT1 expression in PIC-OCT treated cells.* The 661W cells were treated with vehicle (A-C) or 40 µM of PIC-OCT (B-D) and then were fixed and immunostained with anti-SIRT1 antibodies (A, B). The nuclei were visualized by Hoechst dye (C, D). PIC-OCT treatment increases the SIRT1 signal at the nucleus (B-D). Scale bars represent 25 µm.

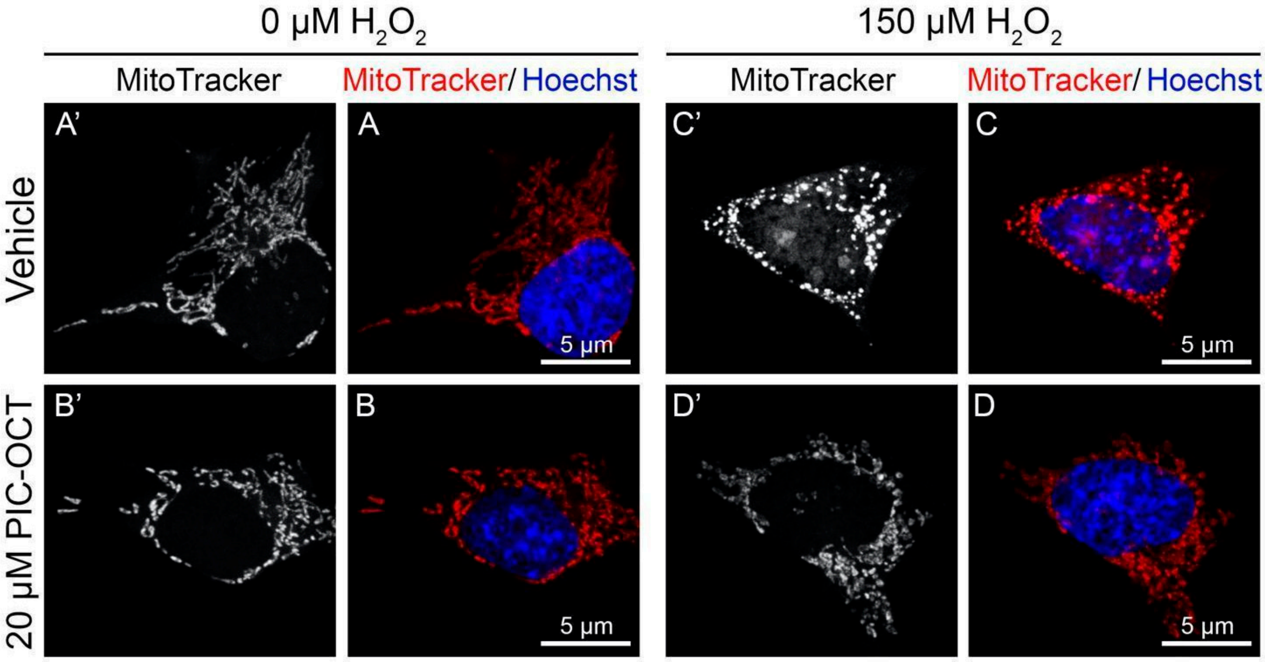

Supplementary Figure S4. *Mitochondrial morphology in PIC-OCT treated cells.* The 661W cells were treated with vehicle (A′-A), 20 μM of PIC-OCT (B′-B), 150 μM H_2O_2 (C′-C) or PIC-OCT + H_2O_2 (D′-D). Then the cells were stained with MitoTracker (red). The nuclei were visualized by Hoechst dye (blue). H_2O_2 produced swelling and fragmentation of the mitochondria and PIC-OCT pretreatment prevented these mitochondrial morphological changes. Scale bars represent 5 μm.
